# Changes in urinary concentrations of contemporary and emerging chemicals in commerce during the COVID-19 pandemic: Insights from the Environmental influences on Child Health Outcomes (ECHO) program

**DOI:** 10.1371/journal.pone.0317358

**Published:** 2025-01-24

**Authors:** Megan E. Romano, Jessie P. Buckley, Xiuhong Li, Julie B. Herbstman, Kurunthachalam Kannan, Sunmi Lee, Susan L. Schantz, Leo Trasande, Margaret R. Karagas, Frederica Perera, for the ECHO Cohort Consortium

**Affiliations:** 1 Department of eEpidemiology, Dartmouth Geisel School of Medicine, Lebanon, NH, United States of America; 2 Department of Epidemiology, University of North Carolina, Chapel Hill, NC, United States of America; 3 Environmental influences on Child Health Outcomes Data Analysis Center, Johns Hopkins University, Baltimore, MD, United States of America; 4 Department of Environmental Health Sciences, Columbia University, New York, NY, United States of America; 5 Department of Pediatrics, New York University, New York, NY, United States of America; 6 Wadsworth Center, New York State Department of Health, Albany, NY, United States of America; 7 Beckman Institute for Advance Science and Technology, University of Illinois Urbana-Champaign, Champaign, IL, United States of America; 8 Department of Pediatrics, Division of Environmental Pediatrics, Department of Population Health, and Department of Environmental Health NYU Grossman School of Medicine, New York, NY, United States of America; 9 New York University Wagner School of Public Service, New York, NY, United States of America; King Faisal Specialist Hospital and Research Center, SAUDI ARABIA

## Abstract

Previous research indicates that the COVID-19 pandemic catalyzed alterations in behaviors that may impact exposures to environmental endocrine-disrupting chemicals. This includes changes in the use of chemicals found in consumer products, food packaging, and exposure to air pollutants. Within the Environmental influences on Child Health Outcomes (ECHO) program, a national consortium initiated to understand the effects of environmental exposures on child health and development, our objective was to assess whether urinary concentrations of a wide range of potential endocrine-disrupting chemicals varied before and during the pandemic. Drawing from three racially, ethnically, and socioeconomically diverse ECHO cohorts, we assessed key differences in urinary chemical concentrations related to environmental exposures through food packaging, use of disinfectants, personal care products and air pollutants using repeated urine samples in a subset of 47 participants, who contributed a urine sample prior to the pandemic (between October 2018 and February 2020) and a subsequent urine sample after the pandemic began (between March 2020 and April 2021). We measured urinary concentrations of analytes across several chemical groups, including polycyclic aromatic hydrocarbons (PAHs), phthalates/alternative plasticizers, synthetic phenols (parabens, bisphenols, triclosan, benzophenones), organophosphate esters (OPEs), insecticides and fungicides. Multivariable linear mixed models accounting for key covariates and clustering within cohort and across repeated samples were used to estimate the change in urinary analyte concentrations across time points. We observed decreases in urinary concentrations of some PAHs, bisphenols, benzophenones, and triclosan, and increases in specific OPEs. These biomarker data mirror some of the behavior changes reported in our prior work and support the observation that the pandemic-related behavior changes lead to alterations in chemical exposures that have been linked to adverse health outcomes.

## Introduction

A “natural experiment” is described as a naturally occurring circumstance in which some individuals within a population experience differential exposure to a potential risk factor without assignment by the investigators [1]. The COVID-19 pandemic represents an unprecedented opportunity to evaluate changes in environmental exposures because of broad-sweeping policies intended to control viral spread. Due to the lockdowns and restrictions imposed by COVID-19, various behaviors shifted, potentially impacting associated environmental exposures. Early phases of the pandemic saw decreased air pollution due to reduced automobile traffic, remote work and school closures [2–5], while heightened use of disinfectant products aimed at reducing viral spread may have increased exposure to some chemicals [6–8]. Additionally, increased emphasis on hand hygiene [9] may have led to greater exposure to chemicals like phthalates found in fragrances [10, 11]. Changes in societal activities, such as limited interactions, restaurant closures, and shifts in the food supply chain, likely contributed to altered dietary habits during the pandemic [12–14]. Some behaviors, like increased home-cooked meals, may have reduced exposure to certain chemicals [15], while others, like heightened snacking or reliance on processed foods, could elevate exposure to endocrine-disrupting chemicals [16–19]. Unintentional consequences of these policies resulted in dynamic behavioral changes that have been linked to changes in chemical exposures, serving as a natural experiment.

The Environmental influences on Child Health Outcomes (ECHO) program, a U.S. wide consortium-based initiative, is well-suited to explore changes in environmental exposures during the COVID-19 pandemic [20]. In our prior work investigating self-reported behavioral changes during the COVID-19 pandemic, we observed noteworthy changes, reported by over a quarter of respondents, including reduced consumption of fast food, decreased use of ultra-processed foods, hair products, and cosmetics [21]. A similar proportion of participants reported an increase in home-cooked meals and the use of antibacterial soaps, liquid soaps, and hand sanitizers [21]. In the present study, we sought to investigate whether urinary concentrations of corresponding environmental endocrine-disrupting chemicals and their metabolites, which serve as biomarkers of systemic exposure, also changed during the pandemic. We measured urinary concentrations of metabolites of polycyclic aromatic hydrocarbons (PAHs), phthalates/alternative plasticizer metabolites, synthetic phenols (parabens, bisphenols, triclosan, benzophenones), organophosphate esters (OPEs), insecticides and fungicides, from repeated urine samples collected among a subset of participants in three ECHO cohorts from before and during the COVID-19 pandemic. We hypothesized that urinary concentrations of the chemicals of interest would mirror the observed behavioral changes in our prior study [21]. Thus, we anticipated decreases in PAHs due to reduced air pollution, and reductions in phthalates/phthalate alternatives and bisphenols due to less consumption of ultra-processed food and more home cooked meals. We also anticipated that urinary concentrations of parabens and benzophenones might decrease due to less frequent use of cosmetics and personal care products, whereas, urinary triclosan might increase due to more use of cleaning products and hand sanitizers. We further planned to examine changes in urinary concentrations of additional endocrine-disrupting chemicals of interest (organophosphate ester flame retardants, insecticides, and fungicides), where previously observed behavior changes to time spent indoors/outdoors were less clear.

## Materials and methods

### Study population

The ECHO Program leverages a combination of extant and new data from participants enrolled during pregnancy from across the United States [20]. We included pregnant people from three ECHO cohorts located in three states (Illinois, New Hampshire, New York) (S1 Table in S1 File). For this study, each cohort contributed repeat urine specimens from up to 20 ECHO participants (total N = 47), with the first sample being collected during pregnancy and on dates before February 29, 2020 (Pre-COVID-19 sample, median date of collection was 9/26/2019 and range was 2/5/2019-2/21/2020) and the second sample collected after delivery and after March 1, 2020 (Peri-COVID sample, median date of collection was 2/8/2021 and range was 3/2/2020-4/22/2021). Study protocols were approved by the local IRB (NYU Langone School of Medicine Institutional Review Board (IRB), University of Illinois Urbana-Champaign IRB, Trustees of Dartmouth College Committee for the Protection of Human Subjects) or central ECHO IRB (WCG™) with written informed consent obtained from participants prior to the start of any cohort-specific research and/or the ECHO-wide Cohort Data Collection Protocol. The work of the ECHO Data Analysis Center is approved through the Johns Hopkins Bloomberg School of Public Health IRB.

### Chemical analysis

The Wadsworth Center-Human Health Exposure Analysis Resource (WC-HHEAR) developed and validated an analytical method for multiple chemical measurements including current use and emerging chemicals of concern [22]. Briefly, pre-COVID and post-COVID urine samples were analyzed simultaneously at WC-HHEAR using solid-phase extraction (SPE) coupled with high-performance liquid chromatography-tandem mass spectrometry (HPLC-MS/MS) [23]. To avoid cohort specific batch effects, samples were randomized such that an equal number of urine samples from each cohort were included in each batch for analysis. Urine samples (0.5 mL) were incubated with β-glucuronidase/arylsulfatase (2000 units) and subjected to ABS Elut NEXUS SPE (Agilent, Santa Clara, CA) prior to analysis by HPLC-MS/MS. A Sciex HPLC system (SCIEX, Redwood City, CA) interfaced with an ABSCIEX QTRAP 5500+ triple quadrupole mass spectrometer (Applied Biosystems, Foster City, CA) with an electrospray ionization source was used in the analysis. The optimal LC-MS/MS conditions and quality assurance protocols are described in detail elsewhere [23].

Limits of detection (LODs) ranged from 0.004 to 4.7 ng/mL across the 104 analytes quantified in at least one sample. Concentration units are ng/mL for all analytes measured. Several classes of chemicals were of specific interest based on previous findings that behavior changes during the COVID-19 pandemic might be related to chemical exposures (S2 Table in S1 File) [21]. These include PAHs, phthalates/alternative plasticizers, OPEs, insecticides, neonicotinoid insecticides, fungicides, bisphenols, benzophenones, and triclosan. To simplify reporting for metabolites of three parent phthalates and the phthalate alternative plasticizer DINCH, we calculated molar sums of metabolites for di-2-ethylhexyl phthalate (DEHP), di-isodecyl phthalate (DiDP), di-(2-propylheptyl) phthalate (DPHP), and di-iso-nonyl-cyclohexane-1,2-dicarboxylic acid (DINCH) as in our prior research [22].

For statistical analyses, concentrations with a signal <LOD were assigned a value of LOD/√2 [24]. We calculated specific gravity (SG) standardized concentrations of urinary analytes as follows: P_c_ = P[SG_ref_-1/SGi_-_1], where P_c_ is the SG-standardized urinary concentration (μg/L), P is the concentration of compound quantified in the urine sample (μg/L), SG_ref_ is the median SG within the study population at the time point of measurement and SG_i_ is the measured SG in each sample [25].

### Participant and urine specimen collection characteristics

Sociodemographic variables assessed at the time of the pre-COVID-19 urine sample included participant age (years; continuous), pre-pregnancy body mass index (BMI; continuous), race/ethnicity (Hispanic, non-Hispanic white, non-Hispanic Black, non-Hispanic Other), highest education attained (less than high school, high school, some college or more), employment status (employed v. not employed), number of people living in the household (continuous), and parity (nulliparous v. parous). Urine specimen collection characteristics included gestational week of collection (for pre-COVID-19 urine samples), urine specific gravity, and calendar season of collection (autumn [September–November], winter [December–February], spring [March–May], summer [June–August]). Duration of time between urine samples (months) was also calculated.

### Statistical analysis

We computed descriptive statistics to summarize participant characteristics and specific gravity standardized urinary analyte concentrations at each time point. Because the distribution of urinary analyte concentrations tended to be right-skewed, we calculated medians and percentiles of interest (25^th^ and 75^th^) at both time points. We calculated the difference in concentrations across time points by subtracting the urinary analyte concentrations at the earlier time point from the concentration observed at the second time point and used a Wilcoxon signed rank test to assess whether there were statistically significant differences in the concentration of urinary analytes across timepoints.

Analytes of interest were retained for further analysis if they were of *a priori* interest based on our prior research [22, 26] and were detected in >20% of urine samples. These included 4 PAHs, 6 phthalates/phthalate alternatives, 4 parabens, 3 OPEs, 3 insecticides/fungicides, 3 bisphenols, 5 benzophenones, and triclosan (S2 Table in S1 File). Specific gravity standardized urinary analytes were log_2_-transformed to diminish the influence of extreme values in regression analyses. We used linear mixed models with random intercepts, accounting for correlations within participant and cohort to examine the change in urinary analyte concentrations across timepoints. All models included a binary indicator for timepoint to estimate the change in urinary analyte concentrations across time points. Due to our small sample size, we strongly favored model parsimony in regression analyses. Our primary model was adjusted for gestational week of pregnancy at pre-COVID-19 sample collection (weeks, centered at 27), age (years, centered at 32 years), duration between urine samples (months, centered at 15), and pre-pregnancy BMI (centered at 27). Covariates were chosen *a priori* to reflect factors anticipated to be associated with urinary concentrations of the analytes of interest, based on observations in our prior work [22].

Additional sensitivity analyses were conducted to assess the influence of inclusion of parity in the multivariable models as well as season of urine collection (at one or both timepoints). Estimates from the main model were also compared to models that were standard deviation (SD) scaled to allow for more direct comparisons across analytes of interest due to variability in the overall range of concentrations observed across the analytes.

**Fig 1:**
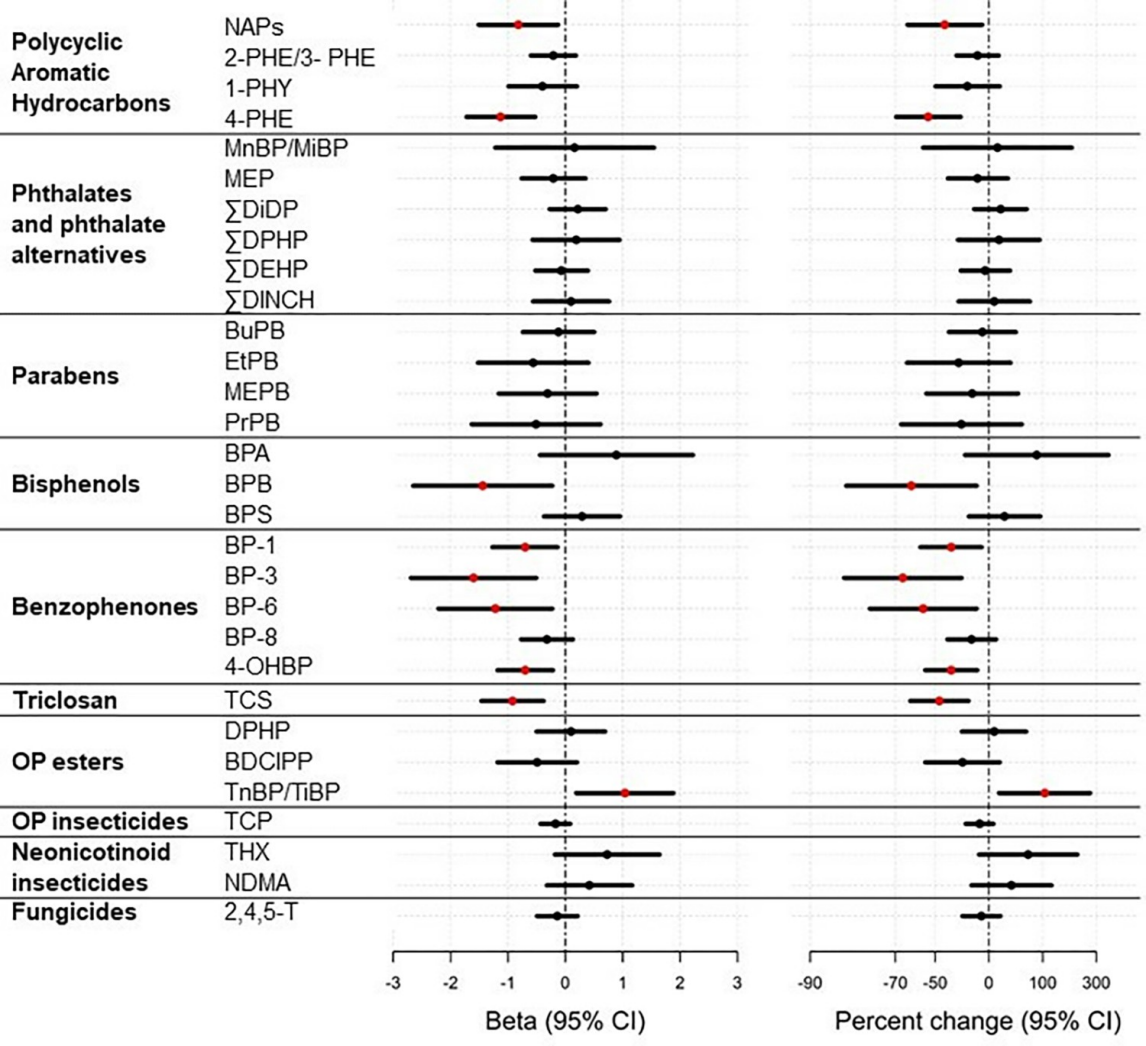
Estimated change in log2-transformed specific gravity standardized chemical analyte concentrations before and during the COVID-19 pandemic and 95% CIs among ECHO participants (n = 47).

We used multiple imputation to account for missing covariates adjusted in the multivariable models, such as pre-pregnancy BMI and parity. Specifically, fully conditional specification (FCS) with a discriminant function method [27] was used to impute binary parous status (n = 4) and FCS predictive mean matching method for imputing the continuous pre-pregnancy BMI (n = 6). Imputation models included the outcome of log_2_-transformed specific gravity standardized urinary analyte concentrations, all covariates in the main and sensitivity analysis models, and cohort-id as a classification variable. The adjusted model results presented in Fig 1 and S3 and S4 Tables in S1 File combine estimates from 25 imputations. We performed statistical analysis using the SAS Enterprise Guide (version 7.13, SAS Institute Inc., Cary NC, USA)analyses and used R version 4.2.2 (R Core Team, Vienna, Austria) to create Fig 1.

## Results

### Participant demographics

On average, our sample of 47 participants was 32.3 years of age (range 21–41 years) and at 17.1 gestational weeks (range 11–36 weeks) of pregnancy at the time of first urine collection (Table 1). They were predominantly non-Hispanic White (74%), with at least some college education (91%), and married or living with a partner (84%). Just under half of participants were nulliparous (40%). On average, the time between repeated urine samples was 14.9 months.

**Table 1:** Demographic and urine specimen collection characteristics of 47 ECHO participants.

*Characteristics*,	
Number of participants	n = 47
	Mean ± SD
Age^a^	32.3 ± 5.5
**Pre-pregnancy BMI**	27.2 ± 8.6
Gestational week of urine sample ^a^	27.1 ± 8.1
**Duration between urine samples (years)**	1.2 ± 0.5
	**n(%)**
**Race/Ethnicity,**	
**Hispanic**	7(15)
**non-Hispanic-White**	35(74)
**non-Hispanic-Other**	5(11)
Maternal education ^b^	
**< High School**	<5
**High School**	<5
**Some college and above**	39(91%)
Employment status (employed)^a^	37(97%)
Married/living with a partner^a^^,^^c^	36(84%)
Number of people in household^a^	
**1–3 people**	18(78%)
**4–6 people**	5(22%)
Nulliparous^a^	17(40%)

^a^ At time of pre-COVID-19 urine sample

^b^Maternal education data representing the highest level of educational attainment was harmonized into three categories: less than high school, high school, or some college and above; n missing = 4

^c^ Marital status was dichotomized as married/living with a partner or not married (widowed; separated; divorced; single, never married; partnered (boyfriend or girlfriend, not living together); n missing = 4

### Analyte concentrations and changes in urinary concentrations of chemicals and metabolites of interest

Among the 30 analytes of interest, most (90%) were detected in >50% of urine samples for at least one time point (Table 2). Nearly all, phthalates and phthalate alternatives [monoethyl phthalate (MEP), and the molar sums of di-iso-decyl phthalate metabolites (∑DiDP), di-(2-propylheptyl) phthalate metabolites (∑DPHP), di-2-ethylhexyl phthalate metabolites (∑DEHP), di-iso-nonyl-cyclohexane-1,2dicarboxylicacid metabolites (∑DINCH)] were detected in every urine sample. However, the composite of mono-isobutyl phthalate mono-n-butyl phthalate (MnBP/MiBP) was infrequently detected (23.4%). Methyl paraben (MePB), diphenyl phosphate (DPHP), 3,5,6-trichloro-2-pyridinol (TCP), and benzophenone-1 (BP-1) were also detectable in all urine samples. Butyl paraben (BuPB, 30.4%-40.4%) and bisphenol A (BPA, 34%-36.2%) were less frequently detected (Table 2).

**Table 2:** Detection frequencies and median concentrations (ng/mL) of specific gravity standardized urinary novel chemicals before and during the COVID-19 pandemic (n = 47).

		Pre-COVID-19^a^		Peri-COVID-19^b^		Difference^c^	
Chemical Class	Analyte	n (%)^d^	Median (25th,75th)	n (%)^d^	Median (25th,75th)	Median (25th,75th)	p-value ^d^
**Polycyclic Aromatic Hydrocarbons**	**NAPs**	43(91.5%)	0.40 (0.25, 0.86)	35(74.5%)	0.37 (0.08, 0.84)	-0.01 (-0.49, 0.17)	0.19
	**2-PHE/3- PHE**	45(95.7%)	0.12 (0.09, 0.21)	39(83%)	0.11 (0.09, 0.19)	0.00 (-0.10, 0.04)	0.47
	**1-PHY**	35(74.5%)	0.06 (0.03, 0.13)	29(61.7%)	0.05 (0.02, 0.10)	-0.01 (-0.06, 0.02)	0.12
	**4-PHE**	34(72.3%)	0.09 (0.03, 0.18)	17(36.2%)	0.04 (0.01, 0.07)	-0.05 (-0.15, 0.00)	<0.01
**Phthalates and phthalate alternatives **	**MnBP/MiBP**	11(23.4%)	0.03 (0.02, 0.10)	11(23.4%)	0.03 (0.02, 0.09)	0.00 (-0.02, 0.05)	0.65
	**MEP**	47(100%)	16.51 (7.57, 60.77)	47(100%)	17.86 (7.23, 32.81)	0.06 (-25.92, 11.65)	0.62
	**∑DiDP**	47(100%)	3.92 (2.14, 6.61)	47(100%)	4.26 (2.96, 8.82)	0.30 (-2.21, 3.45)	0.31
	**∑DPHP**	47(100%)	1.54 (0.99, 4.38)	47(100%)	1.40 (0.84, 5.51)	0.20 (-1.01, 3.08)	0.43
	**∑DEHP**	47(100%)	13.94 (10.72, 23.39)	47(100%)	17.63 (10.66, 28.59)	1.86 (-5.78, 17.14)	0.57
	**∑DINCH**	47(100%)	0.59 (0.27, 1.26)	47(100%)	0.61 (0.28, 1.37)	0.05 (-0.50, 0.40)	0.88
**Parabens **	**BuPB**	19(40.4%)	0.03 (0.02, 0.05)	16(34%)	0.03 (0.02, 0.07)	0.00 (-0.03, 0.03)	0.87
	**EtPB**	45(95.7%)	0.29 (0.19, 3.07)	42(89.4%)	0.31 (0.13, 1.31)	-0.06 (-0.81, 0.24)	0.24
	**MePB**	47(100%)	17.14 (3.71, 73.66)	47(100%)	10.49 (3.58, 47.33)	-0.67 (-27.99, 8.97)	0.62
	**PrPB**	47(100%)	2.29 (0.31, 11.66)	46(97.9%)	0.79 (0.31, 8.51)	-0.15 (-7.07, 0.95)	0.41
**Bisphenols**	**BPA**	16(34%)	0.03 (0.02, 0.38)	17(36.2%)	0.04 (0.02, 3.01)	0.00 (-0.02, 2.17)	0.17
	**BPB**	27(57.4%)	0.26 (0.01, 0.53)	14(29.8%)	0.01 (0.01, 0.37)	-0.03 (-0.50, 0.03)	0.12
	**BPS**	46(97.9%)	0.50 (0.26, 0.95)	43(91.5%)	0.71 (0.35, 1.13)	0.16 (-0.31, 0.63)	0.30
**Benzophenones**	**BP-1**	47(100%)	5.65 (1.51, 17.51)	47(100%)	2.73 (1.30, 10.51)	-1.13 (-5.27, 0.72)	0.03
	**BP-3**	46(97.9%)	36.58 (12.85, 175.35)	43(91.5%)	19.37 (5.21, 39.73)	-10.47 (-117.30, 3.08)	<0.01
	**BP-6**	42(89.4%)	0.46 (0.13, 1.37)	36(76.6%)	0.17 (0.02, 1.28)	-0.19 (-0.93, 0.02)	0.03
	**BP-8**	42(89.4%)	0.15 (0.08, 0.28)	37(78.7%)	0.10 (0.06, 0.22)	-0.03 (-0.14, 0.05)	0.17
	**4-OHBP**	47(100%)	0.14 (0.09, 0.27)	45(95.7%)	0.09 (0.06, 0.17)	-0.04 (-0.18, 0.04)	<0.01
**Triclosan**	**TCS**	47(100%)	0.45 (0.24, 0.88)	46(97.9%)	0.28 (0.14, 0.62)	-0.17 (-0.53, 0.03)	0.01
**Organophosphate esters **	**DPHP**	47(100%)	0.56 (0.37, 1.06)	47(100%)	0.49 (0.32, 1.09)	-0.01 (-0.32, 0.36)	0.95
	**BDCIPP**	40(85.1%)	0.33 (0.14, 1.10)	39(83%)	0.21 (0.11, 0.71)	-0.09 (-0.65, 0.11)	0.02
	**TnBP/TiBP**	23(48.9%)	0.04 (0.02, 0.51)	24(51.1%)	0.09 (0.02, 2.47)	0.00 (-0.04, 0.54)	0.42
**Organophosphate insecticides**	**TCP**	47(100%)	0.67 (0.50, 1.12)	47(100%)	0.67 (0.47, 0.96)	-0.09 (-0.64, 0.20)	0.10
**Neonicotinoid insecticides**	**THX**	22(46.8%)	0.03 (0.01, 0.34)	27(57.4%)	0.16 (0.02, 0.49)	0.01 (-0.09, 0.36)	0.13
	**NDMA**	45(95.7%)	0.28 (0.13, 0.47)	44(93.6%)	0.42 (0.20, 0.80)	0.06 (-0.19, 0.54)	0.29
**Fungicide**	**2,4,5-T**	46(97.9%)	0.28 (0.19, 0.41)	47(100%)	0.25 (0.13, 0.39)	-0.03 (-0.19, 0.13)	0.60

^a^ Urine sample collected before March 1, 2020

^b^ Urine sample collected on or after March 1, 2020

^c^ Peri-COVID-19 urinary concentration minus pre-COVID-19 urinary concentration

^d^ Number of urine samples and percentage with concentrations observed above the limit of detection; ^e^p-value from Wilcoxon signed rank test

We observed small to modest decreases in concentration of specific gravity-standardized 4-hydroxyphenanthrene (4-PHE) [median of the difference between peri-COVID-19 and pre-COVID-19 concentration; p-value from Wilcoxon rank sum test: -0.05 ng/mL; p<0.01], bis(1,3-dichloro-2-propyl) phosphate (BDCIPP) [-0.09 ng/mL; p = 0.02], BP-1 [-1.13ng/mL; p = 0.03], benzophenone-6 (BP-6)[-0.19ng/mL; p = 0.03], 4-hydroxybenzophenone (4-OHBP)[-0.04 ng/mL; p = 0.03], and triclosan (TCS)[-0.17 ng/mL; p<0.01] across time points. A larger decrease was observed for benzophenone-3 (BP-3), with a median decrease in concentration of 10.47 ng/mL (p<0.01) between pre- and peri-COVID-19 samples (Table 2).

Adjusting for gestational week at pre-COVID sample, duration in months between urine samples, maternal age at pre-COVID-19 sample, and maternal pre-pregnancy BMI, we observed decreases in specific-gravity standardized urinary concentrations of some of the PAHs and synthetic phenols, and an increase in the composite measure of tri-n-butyl phosphate and tri-isobutyl phosphate (TnBP/TiBP) across repeated urinary measurements (Fig 1; S3 Table in S1 File). Among the PAHs, we observed a 43% and 54% decrease in the composite of 1-hydroxyphenanthrene and 9-hydroxyphenanthrene (NAPS) (95% CI: -65%, -9%) and 4-PHE (95% CI: -70%, -31%), respectively. Bisphenol-B (BPB) concentration decreased by 63% between the peri- and pre-COVID-19 (95% CI: -84%, -15%). Among the benzophenones, benzophenone-1 (BP-1), benzophonone-3 (BP-3), BP-6, and 4-OHBP decreased between 38% and 67%. Urinary TCS also decreased by 47% between samples (-64%, -23%). Finally, we observed a 106% increase in TnBP/TiBP, though the confidence interval was wide (95% CI: 14, 268). We did not observe meaningful changes in other urinary chemical or metabolite concentrations across the timepoints (Fig 1; S3 Table in S1 File). Further adjustment for parity or season of urine sample did not change the overall pattern of results (S4 Table in S1 File). The pattern of results was similar when examining changes in the SD-scaled urinary analytes with decreases in NAPS, 4-PHE, BPB, BP-1, BP-3, BP-6, 4-OHBP, and TCS ranging from 19% to 31%, and an increase in TnBP/TiBP [22% (4%, 44%)] (S5 Table in S1 File).

## Discussion

The COVID-19 pandemic had widespread impact on daily life and behaviors, including various behaviors that lead to potential shifts in exposures to environmental chemicals. In our study, conducted within the ECHO program, we observed decreasing urinary concentrations of specific PAHs (NAPs, 4-PHE), synthetic phenols (BPB, BP-1, BP-3, BP-5, 4-OHBP, TCS), and increasing urinary concentrations of TnBP/TiBP. Urinary concentrations of phthalate and phthalate alternative metabolites, parabens, organophosphate and neonicotinoid insecticides, or fungicides did not change across pre- and peri-COVID-19 samples.

The observed decreases in specific gravity-standardized urinary concentrations of polycyclic aromatic hydrocarbons (PAHs) align with expectations of reduced air pollution during the pandemic [2, 4, 5]. We observed reductions in urinary NAPs and 4-PHE. Naphthalene and its alkyl derivatives are a subset of PAHs and are common components of fossil fuels, tobacco smoke, and other combustion processes [28]. Likewise, urinary 4-PHE likely reflects exposure to vehicle exhaust or industrial emissions. The decrease in the concentrations of NAPs and 4-PHE in our study suggests a reduction in exposure to PAHs, potentially associated with the decrease in air pollution during the COVID-19 pandemic.

Respondents in our prior study reported changes in behaviors such as decreased consumption of ultra-processed foods and increased home-cooked meals [21]. However, there was little overlap among participants completing the prior survey and participants in the present study, and we did not observe evidence of changes in urinary phthalate metabolite concentrations during the study period. These findings may suggest that there was limited change in exposure to phthalates and phthalate alternatives during the study time frame. Alternatively, this may be in part due to within person variation in urinary phthalate metabolite concentrations, which reflect exposure to phthalates both from dietary and non-dietary sources. While urinary phthalate metabolite concentrations in the National Health and Nutrition Examination Survey participants tend to be higher among individuals consuming more ultra-processed and fast food consumption [15, 18], consumer and personal care products containing fragrance are also an important source of exposure [25, 29]. Among the chemicals of interest, phthalates demonstrate the most within person variability given their short half-lives in the body (hours to days) [30–32]; a single spot urine sample at each time point of interest may be insufficient to identify true shifts in exposure to phthalates [33]. Across study timepoints, there was some evidence of a decrease in urinary BPB, a less common Bisphenol A substitute [34], which may reflect less consumption of canned foods or be explained by the less frequent detection (29.3%) of BPB in peri-COVID-19 samples versus in pre-COVID-19 samples (57.4%). We found decreases in concentrations of several benzophenones, which are used as UV filters in personal care products and sunscreens. This finding was independent of season and could reflect lower sunscreen use or time spent outside. Alternatively, lower benzophenone concentrations could reflect decreasing use of these chemicals over time, which was suggested in our previous study that found decreasing temporal trends in benzophenone concentrations, particularly for BP8 and 4-OHBP, among pregnancy samples collected between 2008 and 2020 [22].

The observed decrease in urinary triclosan ran counter to our expectation that changes in cleaning habits and increased hand hygiene practices [7, 21] might increase systemic exposure to triclosan. In 2016, the Food and Administration banned triclosan as an active ingredient in hand soap [35] and hand sanitizers [36], though dermal absorption may still occur due to its presence in other personal care products (e.g., antiperspirants) or disinfectants [37–39]. Data from NHANES suggest that urinary triclosan concentrations in the U.S. are decreasing overall [40], so this secular trend as triclosan is increasingly phased out of consumer products may be a stronger overall driver of urinary triclosan concentrations than potential behavior changes during the study time frame.

Our observation of increases in urinary TnBP/TiBP warrants further exploration. TnBP and TiBP are used in a wide range of consumer products including paints and textiles. The increase may be explained by the occurrence of OPEs in surgical, KN95, N95 masks [41, 42], resulting in possible inhalation exposure to OPEs when individuals wore masks during the pandemic. The concentrations and profiles of OPEs found in masks were highly variable across studies [41, 42]. The observed increase in urinary TnBP/TiBP emphasizes the complexity of environmental exposures and the need for comprehensive investigations to understand the multifaceted impact of behavioral changes, especially in the presence of a complex risk (environmental chemicals exposures) versus benefit (infection protection) tradeoff.

Our study had several important limitations and notable strengths. First, the sample size for our study was small, though we were able to use multiple imputation for missing covariate data to maximize our sample size for statistical analysis. Second, we relied on a single spot sample of urine at each time point and did not have information about the time of day when samples were collected. Non-persistent chemicals are generally rapidly metabolized and excreted with short biological half-lives (<1 day), exhibiting high intra-individual variability particularly when exposure sources are episodic [43]. Low intra-class correlation coefficients (ICCs) have been reported for bisphenols, high molecular weight phthalates, and other chemicals with dietary sources, while ICCs are in the moderate range for low molecular weight phthalates, OPEs, parabens, and other chemicals found in the indoor environment and personal care products where exposures are more stable over time [44]. These differences in variability of urinary non-persistent chemicals and their metabolites may introduce some measurement error into our exposure assessment. Another notable limitation is that our first samples were obtained during pregnancy and the majority of the second samples were collected after delivery, which may reflect changes to dietary and behavioral patterns following pregnancy [45, 46]. Experimental evidence from animal models also suggests that metabolism of some chemicals, such as phthalates, may also change during pregnancy [47]. Finally, we also cannot rule out the possibility of regression to the mean, thus our findings must be interpreted with caution. Nevertheless, our observations take advantage of the natural experiment provided by the COVID-19 pandemic, allowing us to generate important hypotheses about how changes in behavior during the COVID-19 pandemic may have influenced environmental chemical exposures. These findings are important for contextualizing the findings of other observational studies about environmental chemical exposures undertaken during this same time period.

## Conclusions

The COVID-19 pandemic served as a natural experiment, offering insights into the intricate interplay between behavioral changes and environmental exposures. The observed shifts in urinary concentrations of environmental endocrine-disrupting chemicals provide evidence of the dynamic relationship between public health interventions and consequences on environmental exposures. The observed changes in urinary concentrations of environmental chemicals also provide insights into the intricate relationship between behaviors and environmental exposures, which may inform potential health outcomes. These findings contribute to the growing body of literature on the environmental impacts of the pandemic and highlight the need for continued research to inform public health strategies and mitigate potential adverse effects of environmental exposures on health.

## Supporting information

S1 File Supplemental material. Supplemental tables and appendix.
(PDF)
