# Supplementary material for: Changes in urinary concentrations of contemporary and emerging chemicals in commerce during the COVID-19 pandemic: Insights from the Environmental influences on Child Health Outcomes (ECHO) program
Source: PLoS One. 2025 Jan 24;20(1):e0317358. doi: 10.1371/journal.pone.0317358 (PMC11759379; doi:10.1371/journal.pone.0317358)
Supplement: S1 File — Supplemental tables and appendix. (PDF) [file pone.0317358.s001.pdf]

**Supplemental Material**

# Changes in Urinary Concentrations of Contemporary and Emerging Chemicals in Commerce during the COVID-19 Pandemic: insights from the Environmental influences on Child Health Outcomes (ECHO) Program

Megan E. Romano, Jessie P. Buckley, Xiuhong Li, Julie B. Herbstman, Kurunthachalam Kannan, Sunmi Lee, Susan Schantz, Leo Trasande, Margaret R. Karagas, and Frederica Perera on behalf of the program collaborators for Environmental influences on Child Health Outcomes

## Table of Contents

Table S1: Characteristics of Participating ECHO Pregnancy Cohorts (n=3) ...........................................……………ii

Table S2: Abbreviations and Limits of Detection (LOD) for Main Analytes of Interest...........................…...………iii

Table S3: Estimated change in log2-transformed specific gravity standardized chemical analyte concentrations before and during the COVID-19 pandemic and 95% CIs among ECHO Participants (n=47) ………………………………. iv

Table S4: Unadjusted and adjusted models estimating change in log2-transformed specific gravity standardized chemical analyte concentrations before and during the COVID-19 pandemic and 95% CIs among ECHO Participants (n=47)...........................................................................................................................................….v

Table S5. Estimated change in standard deviation scaled log2-transformed specific gravity standardized chemical analyte concentrations before and during the COVID-19 pandemic and 95% CIs among ECHO Participants (n=47) …………vi

Appendix - ECHO Consortium Members…………………………………………………………………………..vii

**Table S1: Characteristics of Participating ECHO Pregnancy Cohorts (n=3)**

| Cohort name | Location(s) (years of cohort enrollment ) | Recruitment timing and eligibility criteria | Cohort features | n present study |
|---|---|---|---|---|
| **Children's Health and the Environment Study (CHES) (1)** | New York City Metropolitan area, New York (2016-present) | Pregnant people were recruited from three New York University Grossman School of Medicine affiliate hospitals. Eligibility criteria included ≥ 18 years of age, <18 weeks pregnant, had a pregnancy that was not medically threatened, intention to deliver at one of the study hospitals, and fluency in English, Spanish, or Chinese. | This urban cohort is multiracial, multiethnic, and has substantial socioeconomic diversity. | 20 |
| **Illinois Kids Developmental Study (IKIDS) (2)** | Champaign Urbana, Illinois (2013-present) | Pregnant people were recruited from two obstetric clinics at their first prenatal visit and enrolled at 10–14 weeks of gestation. Eligibility criteria included 18–40 years of age, not caring multiples, not in a high-risk pregnancy, fluent in English, not planning to leave the area before the child's first birthday. | Cohort is from a mid-size mid-western college town, predominantly non-Hispanic White and college-educated. | 17 |
| **New Hampshire Birth Cohort Study (NHBCS) (3)** | Concord and Lebanon, New Hampshire (2009-present) | Pregnant people 18–45 years of age were recruited at either ~12 weeks or ~ 24– 28 weeks of gestation from prenatal clinics in New Hampshire. Eligibility criteria included English literacy, use of a private, unregulated water system at home, and a singleton pregnancy. | Participants are representative of the vulnerable group of rural pregnant people and are primarily non-Hispanic White, reflective of the underlying population of the region. | 10 |


(1) Trasande, L., Ghassabian, A., Kahn, L.G. *et al.* The NYU Children's Health and Environment Study. *Eur J Epidemiol* **35**, 305–320 (2020).

(2) Eick, S. M.; Enright, E. A.; Geiger, S. D.; Dzwilewski, K. L. C.; DeMicco, E.; Smith, S.; Park, J. S.; Aguiar, A.; Woodruff, T. J.; Morello-Frosch, R.; Schantz, S. L., Associations of Maternal Stress, Prenatal Exposure to Per- and Polyfluoroalkyl Substances (PFAS), and Demographic Risk Factors with Birth Outcomes and Offspring Neurodevelopment: An Overview of the ECHO.CA.IL Prospective Birth Cohorts. Int J Environ Res Public Health 2021, 18, (2).

(3) Gilbert-Diamond, D.; Emond, J. A.; Baker, E. R.; Korrick, S. A.; Karagas, M. R., Relation between in Utero Arsenic Exposure and Birth Outcomes in a Cohort of Mothers and Their Newborns from New Hampshire. Environ Health Perspect 2016, 124, (8), 1299-307.

Table S2: Abbreviations and Limits of Detection (LOD) for Main Analytes of Interest

| | Abbreviation | Analyte full name | LOD |
|---|---|---|---|
| Polycyclic Aromatic Hydrocarbons (PAHs) | **NAPs** | Composite of 1-hydroxyphenanthrene and 9-hydroxyphenanthrene | 0.027 |
| | **2-PHE/3- PHE** | 2-hydroxyphenanthrene + 3-hydroxyphenanthrene | 0.037 |
| | **1-PHY** | 1-hydroxypyrene | 0.020 |
| | **4-PHE** | 4-hydroxyphenanthrene | 0.025 |
| Phthalates and Phthalate Alternatives | **MnBP/MiBP** | Composite of mono-isobutyl phthalate and mono-n-butyl phthalate | 0.032 |
| | **MEP** | Monoethyl phthalate | 0.021 |
| | **∑DiDP** | Molar sum of di-iso-decyl phthalate metabolites | NA |
| | **∑DPHP** | Molar sum of di-(2-propylheptyl) phthalate metabolites | NA |
| | **∑DEHP** | Molar sum of di-2-ethylhexyl phthalate metabolites | NA |
| | **∑DINCH** | Molar sum of di-iso-nonyl-cyclohexane-1,2dicarboxylicacid metabolites | NA |
| Parabens | **BuPB** | Butyl paraben | 0.029 |
| | **EtPB** | Ethyl paraben | 0.024 |
| | **MEPB** | Methyl Paraben | 0.021 |
| | **PrPB** | Propyl paraben | 0.019 |
| Bisphenols | **BPA** | Bisphenol A | 0.027 |
| | **BPB** | Bisphenol B | 0.014 |
| | **BPS** | Bisphenol S | 0.012 |
| Benzophenones | **BP-1** | Benzophenone-1 | 0.023 |
| | **BP-3** | Benzophenone-3 | 0.019 |
| | **BP-6** | Benzophenone-6 | 0.013 |
| | **BP-8** | Benzophenone-8 | 0.033 |
| | **4-OHBP** | 4-hydroxybenzophenone | 0.023 |
| Triclosan | **TCS** | Triclosan | 0.026 |
| Organophosphate Esters (OPEs) | **DPHP** | Diphenyl phosphate | 0.018 |
| | **BDCIPP** | Bis(1,3-dichloro-2-propyl) phosphate | 0.027 |
| | **TnBP/TiBP** | Composite of Tri-n-butyl phosphate and Tri-isobutyl phosphate | 0.031 |
| Organophosphate insecticides | **TCP** | 3,5,6-trichloro-2-pyridinol | 0.004 |
| Neonicotinoid insecticides | **THX** | Thiamethoxam | 0.021 |
| | **NDMA** | N-desmethyl acetamiprid | 0.022 |
| Fungicide | **2,4,5-T** | 2,4,5-Trichlorophenoxyacetic acid | 0.020 |

Table S3: Estimated change in log2-transformed specific gravity standardized chemical analyte concentrations before and during the COVID-19 pandemic and 95% CIs among ECHO Participants (n=47)

| Chemical Class | Analyte | Beta (95% CI)[a] | Percent Change (95% CI) [a,b] |
|---|---|---|---|
| **Polycyclic Aromatic Hydrocarbons** | **NAPs** | **-0.82 (-1.51, -0.13)** | **-43 (-65, -9)** |
| | **2-PHE/3- PHE** | -0.21 (-0.6, 0.18) | -14 (-34, 13) |
| | **1-PHY** | -0.4 (-0.99, 0.2) | -24 (-50, 15) |
| | **4-PHE** | **-1.13 (-1.72, -0.53)** | **-54 (-70, -31)** |
| **Phthalates and phthalate alternatives** | **MnBP/MiBP** | 0.16 (-1.22, 1.54) | 12 (-56, 191) |
| | **MEP** | -0.21 (-0.76, 0.35) | -14 (-41, 27) |
| | **∑DiDP** | 0.22 (-0.27, 0.7) | 16 (-13, 62) |
| | **∑DPHP** | 0.19 (-0.57, 0.94) | 14 (-29, 92) |
| | **∑DEHP** | -0.07 (-0.52, 0.39) | -5 (-30, 31) |
| | **∑DINCH** | 0.1 (-0.56, 0.76) | 7 (-32, 69) |
| **Parabens** | **BuPB** | -0.12 (-0.74, 0.5) | -8 (-40, 41) |
| | **EtPB** | -0.56 (-1.52, 0.4) | -32 (-65, 32) |
| | **MEPB** | -0.31 (-1.16, 0.54) | -19 (-55, 45) |
| | **PrPB** | -0.51 (-1.63, 0.61) | -30 (-68, 53) |
| **Bisphenols** | **BPA** | 0.89 (-0.44, 2.22) | 85 (-24, 366) |
| | **BPB** | **-1.44 (-2.65, -0.23)** | **-63 (-84, -15)** |
| | **BPS** | 0.29 (-0.37, 0.95) | 22 (-19, 93) |
| **Benzophenones** | **BP-1** | **-0.7 (-1.27, -0.14)** | **-38 (-56, -9)** |
| | **BP-3** | **-1.6 (-2.69, -0.51)** | **-67 (-84, -30)** |
| | **BP-6** | **-1.22 (-2.21, -0.23)** | **-57 (-78, -15)** |
| | **BP-8** | -0.32 (-0.77, 0.13) | -20 (-41, 9) |
| | **4-OHBP** | **-0.7 (-1.18, -0.22)** | **-38 (-56, -14)** |
| **Triclosan** | **TCS** | **-0.92 (-1.46, -0.38)** | **-47 (-64, -23)** |
| **Organophosphate esters** | **DPHP** | 0.1 (-0.5, 0.69) | 7 (-29, 61) |
| | **BDCIPP** | -0.49 (-1.18, 0.2) | -29 (-56, 15) |
| | **TnBP/TiBP** | **1.04 (0.19, 1.88)** | **106 (14, 268)** |
| **Organophosphate insecticides** | **TCP** | -0.17 (-0.43, 0.08) | -11 (-26, 6) |
| **Neonicotinoid insecticides** | **THX** | 0.73 (-0.18, 1.64) | 66 (-7, 212) |
| | **NDMA** | 0.42 (-0.32, 1.16) | 34 (-19, 123) |
| **Fungicides** | **2,4,5-T** | -0.14 (-0.49, 0.21) | -9 (-29, 16) |

[a] Adjusted for gestational week at pre-COVID sample (centered at 27 weeks) and duration in months between urine samples (centered at 15 months), maternal age at pre-COVID-19 sample (centered at 32 years) and maternal pre-pregnancy BMI (centered at 27).

[b] Percent change was calculated at $(2^{beta}-1) \times 100$

Table S4: Unadjusted and adjusted models estimating change in log2-transformed specific gravity standardized chemical analyte concentrations before and during the COVID-19 pandemic and 95% CIs among ECHO Participants (n=47)

| Analyte Name | Unadjusted | Model 1 | Model 2 | Model 3 | Model 4 | Model 5 | Model 6 |
|---|---|---|---|---|---|---|---|
| NAPs | **-0.8(-1.57,-0.04)** | **-0.82(-1.53,-0.11)** | **-0.82(-1.51,-0.13)** | **-0.82(-1.51,-0.13)** | **-0.82(-1.51,-0.13)** | **-0.82(-1.51,-0.13)** | **-0.82(-1.51,-0.13)** |
| 2-PHE/3- PHE | -0.2(-0.61,0.2) | -0.21(-0.61,0.2) | -0.21(-0.6,0.18) | -0.21(-0.6,0.18) | -0.21(-0.6,0.18) | -0.21(-0.6,0.19) | -0.21(-0.6,0.19) |
| 1-PHY | -0.39(-1,0.23) | -0.4(-1,0.21) | -0.4(-0.99,0.2) | -0.4(-0.99,0.2) | -0.4(-0.99,0.19) | -0.4(-0.99,0.2) | -0.4(-0.99,0.2) |
| 4-PHE | **-1.12(-1.79,-0.46)** | **-1.14(-1.75,-0.52)** | **-1.13(-1.72,-0.53)** | **-1.13(-1.72,-0.53)** | **-1.13(-1.73,-0.53)** | **-1.13(-1.72,-0.53)** | **-1.13(-1.73,-0.53)** |
| MnBP/MiBP | 0.16(-1.25,1.57) | 0.16(-1.26,1.58) | 0.16(-1.22,1.54) | 0.16(-1.22,1.55) | 0.16(-1.23,1.54) | 0.16(-1.22,1.54) | 0.16(-1.23,1.54) |
| MEP | -0.21(-0.77,0.36) | -0.2(-0.77,0.37) | -0.21(-0.76,0.35) | -0.21(-0.77,0.35) | -0.21(-0.76,0.35) | -0.21(-0.76,0.35) | -0.21(-0.76,0.35) |
| ∑DiDP | 0.22(-0.27,0.71) | 0.22(-0.28,0.71) | 0.22(-0.27,0.7) | 0.22(-0.26,0.7) | 0.22(-0.27,0.7) | 0.22(-0.26,0.69) | 0.22(-0.26,0.69) |
| ∑DPHP | 0.18(-0.6,0.96) | 0.19(-0.59,0.96) | 0.19(-0.57,0.94) | 0.19(-0.57,0.95) | 0.19(-0.58,0.95) | 0.19(-0.56,0.93) | 0.19(-0.56,0.94) |
| ∑DEHP | -0.07(-0.54,0.4) | -0.07(-0.54,0.4) | -0.07(-0.52,0.39) | -0.07(-0.52,0.39) | -0.07(-0.53,0.39) | -0.07(-0.53,0.39) | -0.07(-0.53,0.39) |
| ∑DINCH | 0.1(-0.56,0.77) | 0.1(-0.57,0.77) | 0.1(-0.56,0.76) | 0.1(-0.56,0.75) | 0.1(-0.55,0.76) | 0.1(-0.54,0.75) | 0.1(-0.54,0.74) |
| BuPB | -0.12(-0.75,0.51) | -0.12(-0.75,0.52) | -0.12(-0.74,0.5) | -0.12(-0.74,0.5) | -0.12(-0.74,0.5) | -0.12(-0.74,0.5) | -0.12(-0.74,0.5) |
| EtPB | -0.55(-1.56,0.45) | -0.57(-1.55,0.41) | -0.56(-1.52,0.4) | -0.56(-1.52,0.4) | -0.56(-1.52,0.4) | -0.56(-1.52,0.4) | -0.56(-1.52,0.4) |
| MEPB | -0.31(-1.18,0.57) | -0.31(-1.19,0.56) | -0.31(-1.16,0.54) | -0.31(-1.16,0.54) | -0.31(-1.16,0.54) | -0.31(-1.16,0.54) | -0.31(-1.16,0.54) |
| PrPB | -0.53(-1.67,0.61) | -0.53(-1.69,0.62) | -0.51(-1.63,0.61) | -0.5(-1.62,0.62) | -0.51(-1.63,0.61) | -0.51(-1.63,0.62) | -0.51(-1.63,0.62) |
| DPHP | 0.11(-0.54,0.76) | 0.1(-0.52,0.71) | 0.1(-0.5,0.69) | 0.1(-0.5,0.69) | 0.1(-0.5,0.69) | 0.1(-0.5,0.69) | 0.1(-0.5,0.69) |
| BPA | 0.89(-0.46,2.24) | 0.9(-0.46,2.25) | 0.89(-0.44,2.22) | 0.9(-0.43,2.23) | 0.89(-0.44,2.22) | 0.89(-0.44,2.22) | 0.89(-0.43,2.21) |
| BPB | **-1.42(-2.69,-0.16)** | **-1.44(-2.68,-0.19)** | **-1.44(-2.65,-0.23)** | **-1.44(-2.65,-0.23)** | **-1.44(-2.66,-0.22)** | **-1.44(-2.65,-0.23)** | **-1.44(-2.66,-0.22)** |
| BPS | 0.3(-0.44,1.04) | 0.29(-0.39,0.97) | 0.29(-0.37,0.95) | 0.29(-0.38,0.95) | 0.29(-0.37,0.95) | 0.29(-0.37,0.95) | 0.29(-0.37,0.95) |
| BP-1 | **-0.68(-1.31,-0.05)** | **-0.7(-1.27,-0.12)** | **-0.7(-1.27,-0.14)** | **-0.71(-1.27,-0.14)** | **-0.7(-1.27,-0.13)** | **-0.7(-1.27,-0.14)** | **-0.7(-1.27,-0.14)** |
| BP-3 | **-1.58(-2.75,-0.4)** | **-1.59(-2.71,-0.48)** | **-1.6(-2.69,-0.51)** | **-1.62(-2.71,-0.53)** | **-1.6(-2.69,-0.52)** | **-1.61(-2.69,-0.52)** | **-1.61(-2.69,-0.52)** |
| BP-6 | **-1.23(-2.23,-0.23)** | **-1.23(-2.24,-0.22)** | **-1.22(-2.21,-0.23)** | **-1.22(-2.21,-0.24)** | **-1.22(-2.21,-0.23)** | **-1.23(-2.21,-0.24)** | **-1.22(-2.21,-0.24)** |
| BP-8 | -0.31(-0.79,0.16) | -0.32(-0.78,0.14) | -0.32(-0.77,0.13) | -0.32(-0.77,0.13) | -0.32(-0.77,0.13) | -0.32(-0.77,0.13) | -0.32(-0.77,0.13) |
| 4-OHBP | **-0.68(-1.21,-0.16)** | **-0.69(-1.18,-0.2)** | **-0.7(-1.18,-0.22)** | **-0.7(-1.18,-0.22)** | **-0.7(-1.18,-0.22)** | **-0.7(-1.18,-0.22)** | **-0.7(-1.18,-0.22)** |
| TCS | **-0.91(-1.56,-0.26)** | **-0.92(-1.47,-0.37)** | **-0.92(-1.46,-0.38)** | **-0.93(-1.47,-0.39)** | **-0.92(-1.46,-0.38)** | **-0.92(-1.45,-0.38)** | **-0.92(-1.46,-0.38)** |
| BDCIPP | -0.49(-1.22,0.24) | -0.5(-1.2,0.21) | -0.49(-1.18,0.2) | -0.49(-1.18,0.2) | -0.49(-1.18,0.2) | -0.49(-1.18,0.2) | -0.49(-1.18,0.2) |
| TnBP/TiBP | **1.03(0.15,1.92)** | **1.04(0.17,1.91)** | **1.04(0.19,1.88)** | **1.03(0.18,1.88)** | **1.04(0.2,1.88)** | **1.04(0.2,1.88)** | **1.04(0.21,1.87)** |
| TCP | -0.17(-0.44,0.1) | -0.17(-0.43,0.09) | -0.17(-0.43,0.08) | -0.17(-0.43,0.08) | -0.17(-0.43,0.08) | -0.17(-0.43,0.08) | -0.17(-0.43,0.08) |
| THX | 0.73(-0.2,1.66) | 0.73(-0.21,1.66) | 0.73(-0.18,1.64) | 0.73(-0.18,1.64) | 0.73(-0.18,1.64) | 0.73(-0.19,1.65) | 0.73(-0.18,1.64) |
| NDMA | 0.43(-0.33,1.19) | 0.42(-0.33,1.18) | 0.42(-0.32,1.16) | 0.42(-0.32,1.16) | 0.42(-0.33,1.17) | 0.42(-0.33,1.17) | 0.42(-0.34,1.18) |
| 2,4,5-T | -0.13(-0.51,0.25) | -0.13(-0.5,0.23) | -0.14(-0.49,0.21) | -0.14(-0.49,0.21) | -0.14(-0.49,0.21) | -0.14(-0.49,0.21) | -0.14(-0.49,0.21) |

Model 1 is adjusted for gestational week at pre-COVID sample (centered at 27 weeks) and duration in months between urine samples (centered at 15 months)
Model 2 is adjusted for Model 1 covariates plus maternal age at Pre-COVID sample (centered at 32 years) and maternal pre-pregnancy BMI (centered at 27).
Model 3 is adjusted for Model 2 covariates plus parity (nulliparous v. parous)
Model 4 is adjusted for Model 2 covariates plus season of Pre-COVID urine sample
Model 5 is adjusted for Model 2 covariates plus season of Peri-COVID urine sample
Model 6 is adjusted for Model 2 covariates plus season of Pre-COVID urine sample and season of Post-COVID urine sample

Table S5. Estimated change in standard deviation scaled log2-transformed specific gravity standardized chemical analyte concentrations before and during the COVID-19 pandemic and 95% CIs among ECHO Participants (n=47)

| Chemical Class | Analyte | Beta (95% CI)[a, c] | Percent Change (95% CI)[a, b, c] |
|---|---|---|---|
| **Polycyclic Aromatic Hydrocarbons** | **NAPs** | **-0.39(-0.72, -0.06)** | **-24(-39, -4)** |
| | **2-PHE/3- PHE** | -0.19(-0.54, 0.17) | -12(-31, 12) |
| | **1-PHY** | -0.26(-0.64, 0.13) | -16(-36, 9) |
| | **4-PHE** | **-0.66(-1.01, -0.31)** | **-37(-50, -20)** |
| **Phthalates and phthalate alternatives** | **MnBP/MiBP** | 0.04(-0.29, 0.36) | 3(-18, 29) |
| | **MEP** | -0.11(-0.41, 0.19) | -7(-25, 14) |
| | **∑DiDP** | 0.17(-0.21, 0.54) | 12(-13, 46) |
| | **∑DPHP** | 0.1(-0.3, 0.5) | 7(-19, 42) |
| | **∑DEHP** | -0.06(-0.43, 0.32) | -4(-26, 25) |
| | **∑DINCH** | 0.06(-0.33, 0.45) | 4(-20, 37) |
| **Parabens** | **BuPB** | -0.07(-0.45, 0.3) | -5(-27, 24) |
| | **EtPB** | -0.19(-0.51, 0.13) | -12(-30, 10) |
| | **MEPB** | -0.12(-0.45, 0.21) | -8(-27, 16) |
| | **PrPB** | -0.16(-0.52, 0.19) | -11(-30, 14) |
| **Bisphenols** | **BPA** | 0.27(-0.13, 0.67) | 20(-9, 59) |
| | **BPB** | -0.45(-0.82, -0.07) | **-27(-43, -5)** |
| | **BPS** | 0.16(-0.21, 0.53) | 12(-13, 44) |
| **Benzophenones** | **BP-1** | **-0.31(-0.56, -0.06)** | **-19(-32, -4)** |
| | **BP-3** | **-0.46(-0.77, -0.15)** | **-27(-41, -10)** |
| | **BP-6** | -0.42(-0.77, -0.08) | **-25(-41, -5)** |
| | **BP-8** | -0.23(-0.55, 0.09) | -15(-32, 7) |
| | **4-OHBP** | **-0.53(-0.9, -0.17)** | **-31(-46, -11)** |
| **Triclosan** | **TCS** | **-0.49(-0.78, -0.2)** | **-29(-42, -13)** |
| **Organophosphate esters** | **DPHP** | 0.05(-0.28, 0.39) | 4(-18, 31) |
| | **BDCIPP** | -0.25(-0.59, 0.1) | -16(-34, 7) |
| | **TnBP/TiBP** | **0.29(0.05, 0.53)** | **22(4, 44)** |
| **Organophosphate insecticides** | **TCP** | -0.2(-0.49, 0.1) | -13(-29, 7) |
| **Neonicotinoid insecticides** | **THX** | 0.29(-0.07, 0.66) | 22(-5, 58) |
| | **NDMA** | 0.23(-0.17, 0.64) | 17(-11, 55) |
| **Fungicides** | **2,4,5-T** | -0.15(-0.53, 0.23) | -10(-31, 18) |

[a] Adjusted for gestational week at pre-COVID sample (centered at 27 weeks) and duration in months between urine samples (centered at 15 months), maternal age at pre-COVID-19 sample (centered at 32 years) and maternal pre- pregnancy BMI (centered at 27).
[b] Percent change was calculated at $(2^{beta}-1) \times 100$
[c] Concentrations of log2-transformed urinary analytes were scaled by standard deviation (SD), (SD* (log2 analyte/SD))

| ECHO Consortium Member | Department | Institution | Location (city, state/province, country) | Role or Contribution | ECHO Cohort Study Site or Core Name and Grant Number |
|---|---|---|---|---|---|
| P Brian Smith MD, MPH, MHS | Division of Neonatology, Department of Pediatrics | Duke Clinical Research Institute, Duke University School of Medicine | Durham, North Carolina, USA | ECHO Coordinating Center Principal Investigator | U2COD023375 (Coordinating Center) |
| L Kristin Newby MD, MHS | Division of Cardiology, Department of Medicine | Duke Clinical Research Institute, Duke University School of Medicine | Durham, North Carolina, USA | ECHO Coordinating Center Principal Investigator | U2COD023375 (Coordinating Center) |
| Linda Adair PhD | Department of Nutrition | Gillings School of Global Public Health, University of North Carolina at Chapel Hill | Chapel Hill, North Carolina, USA | ECHO Coordinating Center Principal Investigator | U2COD023375 (Coordinating Center) |
| Lisa P. Jacobson ScD | Department of Epidemiology | Johns Hopkins University, Bloomberg School of Public Health | Baltimore, Maryland, USA | ECHO Data Analysis Center Principal Investigator | U24OD023382 (Data Analysis Center) |
| Diane Catellier DrPH | N/A | Research Triangle Institute | Research Triangle Park, North Carolina, USA | ECHO Data Analysis Center Principal Investigator | U24OD023382 (Data Analysis Center) |
| Monica McGrath ScD | Department of Epidemiology | Johns Hopkins University, Bloomberg School of Public Health | Baltimore, Maryland, USA | ECHO Johns Hopkins University Data Analysis Center Director Co-Investigator | U24OD023382 (Data Analysis Center) |
| Christian Douglas DrPH | N/A | Research Triangle Institute | Research Triangle Park, North Carolina, USA | ECHO RTI Data Analysis Center Director Co-Investigator | U24OD023382 (Data Analysis Center) |
| Priya Duggal PhD | Department of Epidemiology | Johns Hopkins University, Bloomberg School of Public Health | Baltimore, Maryland, USA | ECHO Data Analysis Center Genetics Methods Lead, Co-Investigator | U24OD023382 (Data Analysis Center) |
| Emily Knapp PhD | Department of Epidemiology | Johns Hopkins University, Bloomberg School of Public Health | Baltimore, Maryland, USA | ECHO Data Analysis Center Co-Investigator | U24OD023382 (Data Analysis Center) |
| Amii Kress PhD | Department of Epidemiology | Johns Hopkins University, Bloomberg School of Public Health | Baltimore, Maryland, USA | ECHO Data Analysis Center General Methods Co-Investigator | U24OD023382 (Data Analysis Center) |
| Courtney K. Blackwell PhD | Department of Medical Social Sciences | Feinberg School of Medicine, Northwestern University | Chicago, Illinois, USA | Measurement Core Co-Investigator | U24OD023319 with co-funding from the Office of Behavioral and Social Science Research (Measurement Core) |
| Maxwell A. Mansolf PhD | Department of Medical Social Sciences | Feinberg School of Medicine, Northwestern University | Chicago, Illinois, USA | Measurement Core Co-Investigator | U24OD023319 with co-funding from the Office of Behavioral and Social Science Research (Measurement Core) |

| | | | | | |
|---|---|---|---|---|---|
| Jin-Shei Lai PhD | Department of Medical Social Sciences | Feinberg School of Medicine, Northwestern University | Chicago, Illinois, USA | Measurement Core Co-Investigator | U24OD023319 with co-funding from the Office of Behavioral and Social Science Research (Measurement Core) |
| Emily Ho PhD | Department of Medical Social Sciences | Feinberg School of Medicine, Northwestern University | Chicago, Illinois, USA | Measurement Core Co-Investigator | U24OD023319 with co-funding from the Office of Behavioral and Social Science Research (Measurement Core) |
| David Cella PhD | Department of Medical Social Sciences | Feinberg School of Medicine, Northwestern University | Chicago, Illinois, USA | Measurement Core Principal Investigator | U24OD023319 with co-funding from the Office of Behavioral and Social Science Research (Measurement Core) |
| Richard Gershon PhD | Department of Medical Social Sciences | Feinberg School of Medicine, Northwestern University | Chicago, Illinois, USA | Measurement Core Principal Investigator | U24OD023319 with co-funding from the Office of Behavioral and Social Science Research (Measurement Core) |
| Michelle L. Macy MD | Department of Pediatrics | Feinberg School of Medicine, Northwestern University and Ann & Robert H. Lurie Children's Hospital of Chicago | Chicago, Illinois, USA | Measurement Core Co-Investigator | U24OD023319 with co-funding from the Office of Behavioral and Social Science Research (Measurement Core) |
| Suman R. Das PhD | Division of Infectious Diseases, Department of Medicine | Vanderbilt University Medical Center | Nashville, Tennessee, USA | ECHO Laboratory Core Principal Investigator | U24OD035523 (Lab Core) |
| Jane E. Freedman MD | Division of Cardiovascular Medicine, Department of Medicine | Vanderbilt University Medical Center | Nashville, Tennessee, USA | ECHO Laboratory Core Principal Investigator | U24OD035523 (Lab Core) |
| Simon A. Mallal MBBS | Division of Infectious Diseases, Department of Medicine | Vanderbilt University Medical Center | Nashville, Tennessee, USA | ECHO Laboratory Core Principal Investigator | U24OD035523 (Lab Core) |
| John A. McLean PhD | Department of Chemistry | Vanderbilt University | Nashville, Tennessee, USA | ECHO Laboratory Core Principal Investigator | U24OD035523 (Lab Core) |
| Ravi V. Shah MD | Division of Cardiovascular Medicine, Department of Medicine | Vanderbilt University Medical Center | Nashville, Tennessee, USA | ECHO Laboratory Core Principal Investigator | U24OD035523 (Lab Core) |
| Meghan H. Shilts MHS | Division of Infectious Diseases, Department of Medicine | Vanderbilt University Medical Center | Nashville, Tennessee, USA | ECHO Laboratory Core Principal Investigator Admin Designee | U24OD035523 (Lab Core) |
| Akram N. Alshawabkeh PhD | College of Engineering | Northeastern University | Boston, Massachusetts, USA | ECHO Cohort Study Site Principal Investigator | UG3/UH3OD023251 (Akram Alshawabkeh) |
| Jose F. Cordero MD | College of Public Health, Department of Epidemiology & Biostatistics | University of Georgia | Athens, Georgia; USA | ECHO Cohort Study Site Co-Director | UG3/UH3OD023251 (Akram Alshawabkeh) |
| John Meeker ScD | Environmental Health Sciences, School of Public Health | University of Michigan | Ann Arbor, Michigan; USA | ECHO Cohort Study Site Co-Director | UG3/UH3OD023251 (Akram Alshawabkeh) |
| Leonardo Trasande MD, MPP | Departments of Pediatrics and Population Health | NYU Grossman School of Medicine | New York, New York, USA | ECHO Cohort Study Site Principal Investigator | UG3/UH3OD023305 (Leonardo Trasande) |
| Carlos A. Camargo MD, DrPH | Department of Emergency Medicine | Massachusetts General Hospital, Harvard Medical School | Boston, Massachusetts, USA | ECHO Cohort Study Site Principal Investigator | UG3/UH3OD023253 (Carlos Camargo) |

| | | | | | |
|---|---|---|---|---|---|
| Kohei Hasegawa MD, PhD | Department of Emergency Medicine | Massachusetts General Hospital, Harvard Medical School | Boston, Massachusetts, USA | ECHO Cohort Study Site Co-Investigator | UG3/UH3OD023253 (Carlos Camargo) |
| Zhaozhong Zhu ScD | Department of Emergency Medicine | Massachusetts General Hospital, Harvard Medical School | Boston, Massachusetts, USA | ECHO Cohort Study Site Co-Investigator | UG3/UH3OD023253 (Carlos Camargo) |
| Ashley F. Sullivan MS, MPH | Department of Emergency Medicine | Massachusetts General Hospital, Harvard Medical School | Boston, Massachusetts, USA | ECHO Cohort Study Site Award Project Director | UG3/UH3OD023253 (Carlos Camargo) |
| Dana Dabelea MD, PhD | Lifecourse Epidemiology of Adiposity and Diabetes (LEAD) Center | University of Colorado Anschutz Medical Campus | Aurora, Colorado, USA | ECHO Cohort Study Site Principal Investigator | UG3/UH3OD023248 and UG3OD035526 (Dana Dabelea) |
| Wei Perng PhD, MPH | Lifecourse Epidemiology of Adiposity and Diabetes (LEAD) Center | University of Colorado Anschutz Medical Campus | Aurora, Colorado, USA | ECHO Cohort Study Site Principal Investigator | UG3/UH3OD023248 (Dana Dabelea) |
| Traci A. Bekelman PhD, MPH | Lifecourse Epidemiology of Adiposity and Diabetes (LEAD) Center | University of Colorado Anschutz Medical Campus | Aurora, Colorado, USA | ECHO Cohort Study Site Principal Investigator | UG3/UH3OD023248 (Dana Dabelea) |
| Greta Wilkening PhD, MPH | Lifecourse Epidemiology of Adiposity and Diabetes (LEAD) Center | University of Colorado Anschutz Medical Campus | Aurora, Colorado, USA | ECHO Cohort Study Site Co-Investigator | UG3/UH3OD023248 (Dana Dabelea) |
| Sheryl Magzamen PhD | Environmental and Radiological Health Sciences | Colorado School of Public Health, Colorado State University | Fort Collins, Colorado, USA | ECHO Cohort Study Site Co-Investigator | UG3OD035526 (Dana Dabelea) |
| Brianna F. Moore PhD, MS | Lifecourse Epidemiology of Adiposity and Diabetes (LEAD) Center | University of Colorado Anschutz Medical Campus | Aurora, Colorado, USA | ECHO Cohort Study Site Principal Investigator | UG3OD035526 (Dana Dabelea) |
| Anne P. Starling PhD | Epidemiology | University of North Carolina at Chapel Hill | Chapel Hill, North Carolina, USA | ECHO Cohort Study Site Principal Investigator | UG3OD035526 (Dana Dabelea) |
| Deborah J. Rinehart PhD | Center for Health Systems Research | Denver Health and Hospital Authority | Denver, Colorado, USA | ECHO Cohort Study Site Co-Investigator | UG3OD035526 (Dana Dabelea) |
| Daphne Koinis Mitchell Ph.D | Department of Pediatrics | Rhode Island Hospital, The Alpert Medical School of Brown University | Providence, Rhode Island, USA | ECHO Cohort Study Site Principal Investigator | UG3/UH3OD023313 (Daphne Koinis Mitchell) |
| Viren D'Sa MD | Department of Pediatrics | Rhode Island Hospital, The Alpert Medical School of Brown University | Providence, Rhode Island, USA | ECHO Cohort Study Site Principal Investigator | UG3/UH3OD023313 (Daphne Koinis Mitchell) |
| Sean C.L. Deoni PhD | Division of Gender Equality, Maternal, Newborn & Child Health Discovery & Tools Team | Bill & Melinda Gates Foundation | Seattle, Washington, USA | ECHO Cohort Study Site Principal Investigator | UG3/UH3OD023313 (Daphne Koinis Mitchell) |
| Hans-Georg Mueller PhD | Department of Statistics | University of California, Davis | Davis, California, USA | ECHO Cohort Study Site Co-Investigator | UG3/UH3OD023313 (Daphne Koinis Mitchell) |
| Cristiane S. Duarte PhD, MPH | Division of Child and Adolescent Psychiatry | Columbia University - NYSPI | New York, New York, USA | ECHO Cohort Study Site Principal Investigator | UH3OD023328 (Cristiane Duarte) |
| Catherine Monk PhD | Department of Obstetrics & Gynecology | Columbia University - NYSPI | New York, New York, USA | ECHO Cohort Study Site Principal Investigator | UH3OD023328 (Cristiane Duarte) |
| Glorisa Canino PhD | Behavioral Sciences Research Institute | University of Puerto Rico, School of Medicine | Rio Piedras, Puerto Rico | ECHO Cohort Study Site Principal Investigator | UH3OD023328 (Cristiane Duarte) |
| Jonathan Posner MD | Child & Family Mental Health & Community Psychiatry Division | Duke University School of Medicine, Duke Psychiatry & Behavioral Sciences | Durham, North Carolina, USA | ECHO Cohort Study Site Principal Investigator | UH3OD023328 (Cristiane Duarte) |

| | | | | | |
|---|---|---|---|---|---|
| Tenneill Murray MPH | Division of Child and Adolescent Psychiatry | Columbia University - NYSPI | New York, New York, USA | ECHO Cohort Study Site Co-Director | UH3OD023328 (Cristiane Duarte) |
| Claudia Lugo-Candelas PhD | Division of Child and Adolescent Psychiatry | Columbia University - NYSPI | New York, New York, USA | ECHO Cohort Study Site Principal Investigator | UH3OD023328 (Cristiane Duarte) |
| Anne L. Dunlop MD, MPH | Department of Gynecology and Obstetrics | Emory University School of Medicine | Atlanta, Georgia, USA | ECHO Cohort Study Site Principal Investigator | UH3OD023318 (Anne Dunlop) |
| Patricia A. Brennan PhD | Department of Psychology | Emory University | Atlanta, Georgia, USA | ECHO Cohort Study Site Principal Investigator | UH3OD023318 (Anne Dunlop) |
| Christine Hockett PhD | N/A; Department of Pediatrics | Avera Research Institute; University of South Dakota School of Medicine | Rapid City, South Dakota, USA; Sioux Falls, South Dakota, USA | ECHO Cohort Study Site Principal Investigator | UG3/UH3OD023279 (Amy Elliott) |
| Amy Elliott PhD | N/A; Department of Pediatrics | Avera Research Institute ; University of South Dakota School of Medicine | Sioux Falls, South Dakota, USA | ECHO Cohort Study Site Principal Investigator | UG3/UH3OD023279 (Amy Elliott) |
| Assiamira Ferrara MD, PhD | Division of Research | Kaiser Permanente Northern California | Oakland, California, USA | ECHO Cohort Study Site Principal Investigator | UG3/UH3OD023289 (Assiamira Ferrara) |
| Lisa A. Croen PhD | Division of Research | Kaiser Permanente Northern California | Oakland, California, USA | ECHO Cohort Study Site Principal Investigator | UG3/UH3OD023342 (Kristen Lyall), UG3/UH3OD023290 (Julie Herbstman) |
| Monique M. Hedderson PhD | Division of Research | Kaiser Permanente Northern California | Oakland, California, USA | ECHO Cohort Study Site Principal Investigator | UG3/UH3OD023289 (Assiamira Ferrara), UG3OD035540 (Monique Marie Hedderson) |
| John Ainsworth PhD | Centre for Health Informatics | University of Manchester | Manchester, United Kingdom | ECHO Cohort Study Site Principal Investigator | UG3/UH3OD023282 (James Gern) |
| Leonard B. Bacharier MD | Department of Pediatrics, Monroe Carell Jr Children’s Hospital at Vanderbilt | Vanderbilt University Medical Center | Nashville, Tennessee, USA | ECHO Cohort Study Site Principal Investigator | UG3/UH3OD023282 (James Gern) |
| Casper G. Bendixsen PhD | National Farm Medicine Center | Marshfield Clinic Research Institute | Marshfield, Wisconsin, USA | ECHO Cohort Study Site Principal Investigator | UG3/UH3OD023282 (James Gern) |
| James E. Gern MD | Department of Pediatrics | University of Wisconsin School of Medicine and Public Health | Madison, Wisconsin, USA | ECHO Cohort Study Site Principal Investigator | UG3/UH3OD023282 (James Gern), UG3OD035509 (Anne Marie Singh) |
| Diane R. Gold MD | The Channing Division of Network Medicine; Department of Medicine | Brigham and Women’s Hospital; Harvard Medical School | Boston, Massachusetts, USA | ECHO Cohort Study Site Principal Investigator | UG3/UH3OD023282 (James Gern) |
| Tina V. Hartert MD, MPH | Division of Pediatric Allergy, Immunology, and Pulmonary Medicine, Department of Medicine, Department of Pediatrics | Vanderbilt University Medical Center | Nashville, Tennessee, USA | ECHO Cohort Study Site Principal Investigator | UG3/UH3OD023282 (James Gern), UG3OD035516 and UG3OD035517 (Tina Hartert) |
| Daniel J. Jackson MD | Department of Pediatrics | University of Wisconsin School of Medicine and Public Health | Madison, Wisconsin, USA | ECHO Cohort Study Site Principal Investigator | UG3/UH3OD023282 (James Gern) |
| Christine C. Johnson PhD | Department of Public Health Sciences | Henry Ford Health | Detroit, Michigan, USA | ECHO Cohort Study Site Principal Investigator | UG3/UH3OD023282 (James Gern), UG3OD035518 (Jennifer Straughen) |
| Christine L.M. Joseph PhD | Department of Public Health Sciences | Henry Ford Health | Detroit, Michigan, USA | ECHO Cohort Study Site Principal Investigator | UG3/UH3OD023282 (James Gern) |
| Meyer Kattan MD | Department of Pediatrics | Columbia University Medical Center | New York, New York, USA | ECHO Cohort Study Site Principal Investigator | UG3/UH3OD023282 (James Gern) |

| | | | | | |
|---|---|---|---|---|---|
| Gurjit K. Khurana Hershey MD, PhD | Division of Asthma Research | Cincinnati Children's Hospital Medical Center | Cincinnati, Ohio, USA | ECHO Cohort Study Site Principal Investigator | UG3/UH3OD023282 (James Gern) |
| Robert F. Lemanske, Jr. MD | Department of Pediatrics | University of Wisconsin School of Medicine and Public Health | Madison, Wisconsin, USA | ECHO Cohort Study Site Principal Investigator | UG3/UH3OD023282 (James Gern) |
| Susan V. Lynch PhD | Department of Medicine | University of California | San Francisco, California, USA | ECHO Cohort Study Site Principal Investigator | UG3/UH3OD023282 (James Gern) |
| Rachel L. Miller MD | Department of Medicine; Division of Clinical Immunology | Icahn School of Medicine at Mount Sinai | New York, New York, USA | ECHO Cohort Study Site Principal Investigator | UG3/UH3OD023282 (James Gern), UG3/UH3OD023290 (Julie Herbstman) |
| George T. O'Connor MD | Department of Pediatrics | Boston University School of Medicine | Boston, Massachusetts, USA | ECHO Cohort Study Site Principal Investigator | UG3/UH3OD023282 (James Gern) |
| Carole Ober PhD | Department of Human Genetics | University of Chicago | Chicago, Illinois, USA | ECHO Cohort Study Site Principal Investigator | UG3/UH3OD023282 (James Gern), UG3OD035509 (Anne Marie Singh) |
| Dennis Ownby MD | Department of Public Health Sciences | Henry Ford Health | Detroit, Michigan, USA | ECHO Cohort Study Site Principal Investigator | UG3/UH3OD023282 (James Gern) |
| Katherine Rivera-Spoljaric MD | Department of Pediatrics | Washington University School of Medicine | St Louis, Missouri, USA | ECHO Cohort Study Site Principal Investigator | UG3/UH3OD023282 (James Gern), UG3OD035521 (Katherine Rivera-Spoljaric) |
| Patrick H. Ryan PhD | Department of Pediatrics and College of Medicine; Division of Biostatistics and Epidemiology | University of Cincinnati | Cincinnati, Ohio, USA | ECHO Cohort Study Site Principal Investigator | UG3/UH3OD023282 (James Gern), UG3OD035509 (Anne Marie Singh) |
| Christine M. Seroogy MD | Department of Pediatrics | University of Wisconsin School of Medicine and Public Health | Madison, Wisconsin, USA | ECHO Cohort Study Site Principal Investigator | UG3/UH3OD023282 (James Gern) |
| Anne Marie Singh MD | Department of Pediatrics | University of Wisconsin School of Medicine and Public Health | Madison, Wisconsin, USA | ECHO Cohort Study Site Principal Investigator | UG3/UH3OD023282 (James Gern), UG3OD035509 (Anne Marie Singh) |
| Robert A. Wood MD | Department of Pediatrics | Johns Hopkins University School of Medicine | Baltimore, Maryland, USA | ECHO Cohort Study Site Principal Investigator | UG3/UH3OD023282 (James Gern) |
| Edward M. Zoratti MD | Division of Allergy and Clinical Immunology | Henry Ford Health | Detroit, Michigan, USA | ECHO Cohort Study Site Principal Investigator | UG3/UH3OD023282 (James Gern), UG3OD035518 (Jennifer Straughen) |
| Rima Habre ScD, MSc | Department of Population and Public Health Sciences | University of Southern California | Los Angeles, California, USA | ECHO Cohort Study Site Principal Investigator | UH3OD023287 (Carrie Breton) |
| Shohreh Farzan PhD | Department of Population and Public Health Sciences | University of Southern California | Los Angeles, California, USA | ECHO Cohort Study Site Principal Investigator | UH3OD023287 (Carrie Breton) |
| Frank D. Gilliland MD, MPH, PhD | Department of Population and Public Health Sciences | University of Southern California | Los Angeles, California, USA | ECHO Cohort Study Site Principal Investigator | UH3OD023287 (Carrie Breton) |
| Irva Hertz-Picciotto PhD | MIND Institute and Department of Public Health Sciences | University of California, Davis | Davis, California, USA | ECHO Cohort Study Site Principal Investigator | UG3/UH3OD023365 (Irva Hertz-Picciotto), UG3OD035550 (Rebecca Schmidt) |
| Deborah H. Bennett Ph.D | Department of Public Health Sciences | University of California, Davis | Davis, California, USA | ECHO Cohort Study Site Principal Investigator | UG3/UH3OD023365 (Irva Hertz-Picciotto), UG3OD035550 (Rebecca Schmidt) |
| Julie B. Schweitzer Ph.D | Department of Psychiatry and Behavioral Science and the MIND Institute | University of California, Davis | Davis, California, USA | ECHO Cohort Study Site Principal Investigator | UG3/UH3OD023365 (Irva Hertz-Picciotto) |

| | | | | | |
|---|---|---|---|---|---|
| Rebecca J. Schmidt Ph.D | MIND Institute and Department of Public Health Sciences | University of California, Davis | Davis, California, USA | ECHO Cohort Study Site Principal Investigator | UG3/UH3OD023365 (Irva Hertz-Picciotto), UG3/UH3OD023342 (Kristen Lyall), UG3OD035550 (Rebecca Schmidt) |
| Janine M. LaSalle PhD | Medical Microbiology and Immunology; MIND Institute | University of California, Davis | Davis, California, USA | ECHO Cohort Study Site Co-Investigator | UG3/UH3OD023365 (Irva Hertz-Picciotto), UG3OD035550 (Rebecca Schmidt) |
| Alison E. Hipwell PhD, ClinPsyD | Psychiatry and Psychology | University of Pittsburgh | Pittsburgh, Pennsylvania, USA | ECHO Cohort Study Site Principal Investigator | UG3/UH3OD023244 (Alison Hipwell) |
| Kate E. Keenan PhD | Psychiatry and Behavioral Neuroscience | University of Chicago | Chicago, Illinois, USA | ECHO Cohort Study Site Principal Investigator | UG3/UH3OD023244 (Alison Hipwell) |
| Catherine J. Karr MD, MS, PhD | Department of Pediatrics, School of Medicine; Department of Environmental and Occupational Health Sciences; School of Public Health | University of Washington | Seattle, Washington, USA | ECHO Cohort Study Site Principal Investigator | UH3OD023271 and UG3OD035528 (Catherine Karr) |
| Nicole R. Bush PhD | Department of Psychiatry and Behavioral Sciences and Department of Pediatrics, School of Medicine | University of California, San Francisco | San Francisco, California, USA | ECHO Cohort Study Site Principal Investigator | UH3OD023271 (Catherine Karr), UG3OD035519 (Qi Zhao) |
| Kaja Z. LeWinn ScD | Department of Psychiatry and Behavioral Sciences, School of Medicine | University of California, San Francisco | San Francisco, California, USA | ECHO Cohort Study Site Principal Investigator | UH3OD023271 (Catherine Karr), UG3OD035519 (Qi Zhao) |
| Sheela Sathyanarayana MD, MPH | Department of Pediatrics, School of Medicine; Department of Environmental and Occupational Health Sciences, School of Public Health | University of Washington and Seattle Children's Research Institute | Seattle, Washington, USA | ECHO Cohort Study Site Principal Investigator | UH3OD023271 (Catherine Karr), UG3OD035508 (Sheela Sathyanarayana) |
| Qi Zhao MD, PhD | Department of Preventive Medicine | University of Tennessee Health Science Center | Memphis, Tennessee, USA | ECHO Cohort Study Site Principal Investigator | UH3OD023271 (Catherine Karr), UG3OD035519 (Qi Zhao) |
| Frances Tylavsky DrPH, MS | Department of Preventive Medicine | University of Tennessee Health Science Center | Memphis, Tennessee, USA | ECHO Cohort Study Site Principal Investigator | UH3OD023271 (Catherine Karr) |
| Kecia N. Carroll MD, MPH | Department of Pediatrics, Department of Environmental Medicine & Public Health | Icahn School of Medicine at Mount Sinai | New York, New York, USA | ECHO Cohort Study Site Principal Investigator | UH3OD023271 (Catherine Karr), UG3/UH3OD023337 (Rosalind Wright) |
| Christine T. Loftus MS MPH PhD | Department of Environmental and Occupational Health Sciences; School of Public Health | University of Washington | Seattle, Washington, USA | ECHO Cohort Study Site Principal Investigator | UH3OD023271 (Catherine Karr) |
| Leslie D. Leve PhD | Department of Counseling Psychology and Human Services & Prevention Science Institute | University of Oregon | Eugene, Oregon, USA | ECHO Cohort Study Site Principal Investigator | UG3/UH3OD023389 (Leslie Leve) |
| Jody M. Ganiban PhD | Department of Psychological and Behavioral Sciences | George Washington University | Washington, DC, USA | ECHO Cohort Study Site Principal Investigator | UG3/UH3OD023389 (Leslie Leve) |
| Jenae M. Neiderhiser PhD | Department of Psychology | Penn State University | University Park, Pennsylvania, USA | ECHO Cohort Study Site Principal Investigator | UG3/UH3OD023389 (Leslie Leve) |
| Scott T. Weiss MD | Channing Division of Network Medicine, Department of Medicine | Brigham and Women's Hospital and Harvard Medical School | Boston, Massachusetts, USA | ECHO Cohort Study Site Principal Investigator | UH3OD023268 (Scott Weiss) |

| | | | | | |
|---|---|---|---|---|---|
| Augusto A. Litonjua MD | Pediatric Pulmonary Division, Department of Pediatrics | Golisano Children's Hospital, University of Rochester | Rochester, New York, USA | ECHO Cohort Study Site Principal Investigator | UH3OD023268 (Scott Weiss) |
| Cindy T. McEvoy MD, MCR | Division of Neonatology, Department of Pediatrics | Oregon Health & Science University | Portland, Oregon, USA | ECHO Cohort Study Site Principal Investigator | UG3/UH3OD023288 (Cynthia McEvoy) |
| Eliot R. Spindel MD, PhD | Division of Neuroscience | Oregon National Primate Research Center | Beaverton, Oregon, USA | ECHO Cohort Study Site Principal Investigator | UG3/UH3OD023288 (Cynthia McEvoy) |
| Robert S. Tepper MD, PhD | Division of Pediatric Pulmonology, Department of Pediatrics | Indiana School of Medicine | Indianapolis, Indiana, USA | ECHO Cohort Study Site Co-Investigator | UG3/UH3OD023288 (Cynthia McEvoy) |
| Craig J. Newschaffer PhD | College of Health and Human Development | Penn State | State College, Pennsylvania, USA | ECHO Cohort Study Site Principal Investigator | UG3/UH3OD023342 (Kristen Lyall) |
| Kristen Lyall ScD | AJ Drexel Autism Institute | Drexel University | Philadelphia, Pennsylvania, USA | ECHO Cohort Study Site Principal Investigator | UG3/UH3OD023342 (Kristen Lyall) |
| Heather E. Volk PhD | Mental Health | Johns Hopkins University | Baltimore, Maryland, USA | ECHO Cohort Study Site Principal Investigator | UG3/UH3OD023342 (Kristen Lyall) |
| Rebecca Landa PhD | Department of Psychiatry and Behavioral Sciences | Center for Autism and Related Disorders, Kennedy Krieger Institute, Johns Hopkins University | Baltimore, Maryland, USA | ECHO Cohort Study Site Co-Investigator | UG3/UH3OD023342 (Kristen Lyall) |
| Sally Ozonoff PhD | MIND Institute, Department of Psychiatry | University of California Davis | Sacramento, California, USA | ECHO Cohort Study Site Co-Investigator | UG3/UH3OD023342 (Kristen Lyall) |
| Joseph Piven MD | Department of Psychiatry | University of North Carolina | Chapel Hill, North Carolina, USA | ECHO Cohort Study Site Co-Investigator | UG3/UH3OD023342 (Kristen Lyall) |
| Heather Hazlett PhD | Department of Psychiatry | University of North Carolina | Chapel Hill, North Carolina, USA | ECHO Cohort Study Site Co-Investigator | UG3/UH3OD023342 (Kristen Lyall) |
| Juhi Pandey PhD | Center for Autism Research | Children's Hospital of Philadelphia | Philadelphia, Pennsylvania, USA | ECHO Cohort Study Site Co-Investigator | UG3/UH3OD023342 (Kristen Lyall) |
| Robert Schultz PhD | Center for Autism Research | Children's Hospital of Philadelphia | Philadelphia, Pennsylvania, USA | ECHO Cohort Study Site Co-Investigator | UG3/UH3OD023342 (Kristen Lyall) |
| Steven Dager PhD | Department of Radiology | University of Washington | Seattle, Washington, USA | ECHO Cohort Study Site Co-Investigator | UG3/UH3OD023342 (Kristen Lyall) |
| Kelly Botteron PhD | Department of Psychiatry | Washington University | St Louis, Missouri, USA | ECHO Cohort Study Site Co-Investigator | UG3/UH3OD023342 (Kristen Lyall) |
| Daniel Messinger PhD | Department of Psychology | University of Miami | Miami, Florida, USA | ECHO Cohort Study Site Co-Investigator | UG3/UH3OD023342 (Kristen Lyall) |
| Wendy Stone PhD | Department of Psychology | University of Washington | Seattle, Washington, USA | ECHO Cohort Study Site Co-Investigator | UG3/UH3OD023342 (Kristen Lyall) |
| Jennifer Ames PhD | Kaiser Permanente Division of Research | Kaiser Permanente | Oakland, California, USA | ECHO Cohort Study Site Co-Investigator | UG3/UH3OD023342 (Kristen Lyall) |
| Thomas G. O'Connor PhD | Departments of Psychiatry, Neuroscience, Obstetrics and Gynecology | University of Rochester | Rochester, New York, USA | ECHO Cohort Study Site Principal Investigator | UG3/UH3OD023349 (Thomas O'Connor) |
| Richard K. Miller PhD | Departments of Obstetrics and Gynecology | University of Rochester | Rochester, New York, USA | ECHO Cohort Study Site Principal Investigator | UG3/UH3OD023349 (Thomas O'Connor) |
| Emily Oken MD, MPH | Division of Chronic Disease Research Across the Lifecourse, Department of Population Medicine | Harvard Pilgrim Health Care Institute and Harvard Medical School | Boston, Massachusetts, USA | ECHO Cohort Study Site Principal Investigator | UH3OD023286 and UG3OD035533 (Emily Oken) |
| Michele R. Hacker ScD | Department of Obstetrics and Gynecology | Beth Israel Deaconess Medical Center | Boston, Massachusetts, USA | ECHO Cohort Study Site Principal Investigator | UG3OD035533 (Emily Oken) |

| | | | | | |
|---|---|---|---|---|---|
| Tamarra James-Todd PhD | Department of Environmental Health | Harvard Chan School of Public Health | Boston, Massachusetts, USA | ECHO Cohort Study Site Principal Investigator | UG3OD035533 (Emily Oken) |
| T. Michael O'Shea, Jr. MD, MPH | Division of Neonatology, Department of Pediatrics | University of North Carolina School of Medicine | Chapel Hill, North Carolina, USA | ECHO Cohort Study Site Principal Investigator | UG3/UH3OD023348 (Mike O’Shea), UH3OD023347 (Barry Lester) |
| Rebecca C. Fry PhD | Department of Environmental Sciences and Engineering | University of North Carolina Gillings School of Global Public Health | Chapel Hill, North Carolina, USA | ECHO Cohort Study Site Principal Investigator | UG3/UH3OD023348 (Mike O’Shea) |
| Jean A. Frazier MD | EK Shriver Center and Psychiatry | UMASS Chan Medical School | Worcster, Massachusetts, USA | ECHO Cohort Study Site Co-Investigator | UG3/UH3OD023348 (Mike O’Shea) |
| Rachana Singh MD, MS | Department of Pediatrics | Tufts University School of Medicine | Boston, Massachusetts, USA | ECHO Cohort Study Site Co-Investigator | UG3/UH3OD023348 (Mike O’Shea) |
| Caitlin Rollins MD, SM | Department of Neurology | Harvard Medical School | Boston, Massachusetts, USA | ECHO Cohort Study Site Co-Investigator | UG3/UH3OD023348 (Mike O’Shea) |
| Angela Montgomery MD | Division of Neonatology, Department of Pediatrics | Yale School of Medicine | New Haven, Connecticut, USA | ECHO Cohort Study Site Co-Investigator | UG3/UH3OD023348 (Mike O’Shea) |
| Ruben Vaidya MD | Department of Pediatrics | University of Massachusetts Chan Medical School-Baystate | Springfield, Massachusetts, USA | ECHO Cohort Study Site Co-Investigator | UG3/UH3OD023348 (Mike O’Shea) |
| Robert M. Joseph PhD | Department of Anatomy & Neurobiology | Boston University Chobanian & Avedisian School of Medicine | Boston, Massachusetts, USA | ECHO Cohort Study Site Co-Investigator | UG3/UH3OD023348 (Mike O’Shea) |
| Lisa K. Washburn MD | Pediatrics | Wake Forest School of Medicine | Winston-Salem, North Carolina, USA | ECHO Cohort Study Site Co-Investigator | UG3/UH3OD023348 (Mike O’Shea) |
| Semsa Gogcu MD, MPH | Section of Neonatology, Department of Pediatrics; Department of Pediatrics | Wake Forest School of Medicine; Wake Forest University School of Medicine/Atrium Health Wake Forest | Winston-Salem, North Carolina, USA | ECHO Cohort Study Site Co-Investigator | UG3/UH3OD023348 (Mike O’Shea), UG3OD035513 (Annemarie Stroustrup), UH3OD023320 (Judy Aschner) |
| Kelly Bear DO | Section of Neonatology, Department of Pediatrics | ECU Health | Greenville, North Carolina, USA | ECHO Cohort Study Site Co-Investigator | UG3/UH3OD023348 (Mike O’Shea) |
| Julie V. Rollins MA | Division of Neonatology, Department of Pediatrics | University of North Carolina School of Medicine | Chapel Hill, North Carolina, USA | ECHO Cohort Study Site Award Project Director | UG3/UH3OD023348 (Mike O’Shea) |
| Stephen R. Hooper PhD | Department of Health Sciences | School of Medicine, University of North Carolina at Chapel Hill | Chapel Hill, North Carolina, USA | ECHO Cohort Study Site Co-Investigator | UG3/UH3OD023348 (Mike O’Shea) |
| Genevieve Taylor MD | Pediatrics | School of Medicine, University of North Carolina at Chapel Hill | Chapel Hill, North Carolina, USA | ECHO Cohort Study Site Co-Investigator | UG3/UH3OD023348 (Mike O’Shea) |
| Wesley Jackson MD, MPH | Division of Neonatology, Department of Pediatrics | University of North Carolina School of Medicine | Chapel Hill, North Carolina, USA | ECHO Cohort Study Site Co-Investigator | UG3/UH3OD023348 (Mike O’Shea) |
| Amanda Thompson PhD | Department of Anthropology, Department of Nutrition | University of North Carolina at Chapel Hill; Gillings School of Global Public Health, University of North Carolina at Chapel Hill | Chapel Hill, North Carolina, USA | ECHO Cohort Study Site Co-Investigator | UG3/UH3OD023348 (Mike O’Shea) |

| | | | | | |
|---|---|---|---|---|---|
| Julie Daniels PhD | Epidemiology and Maternal and Child Health | University of North Carolina at Chapel Hill; Gillings School of Global Public Health, University of North Carolina at Chapel Hill School of Medicine, | Chapel Hill, North Carolina, USA | ECHO Cohort Study Site Co-Investigator | UG3/UH3OD023348 (Mike O'Shea) |
| Michelle Hernandez MD | Pediatrics | University of North Carolina at Chapel Hill | Chapel Hill, North Carolina, USA | ECHO Cohort Study Site Co-Investigator | UG3/UH3OD023348 (Mike O'Shea) |
| Kun Lu PhD | Environmental Sciences and Engineering | Gillings School of Global Public Health, University of North Carolina at Chapel Hill | Chapel Hill, North Carolina, USA | ECHO Cohort Study Site Co-Investigator | UG3/UH3OD023348 (Mike O'Shea) |
| Michael Msall MD | Kennedy Research Center on Intellectual and Neurodevelopmental Disabilities | University of Chicago Medicine: Comer Children's Hospital | Chicago Illinois, USA | ECHO Cohort Study Site Co-Investigator | UG3/UH3OD023348 (Mike O'Shea) |
| Madeleine Lenski MSPH | Department of Epidemiology and Biostatistics | Michigan State University | East Lansing, Michigan, USA | ECHO Cohort Study Site Co-Investigator | UG3/UH3OD023348 (Mike O'Shea) |
| Rawad Obeid MD | Pediatrics | Beaumont Hospital | Royal Oak, Michigan, USA | ECHO Cohort Study Site Co-Investigator | UG3/UH3OD023348 (Mike O'Shea) |
| Steven L. Pastyrnak PhD | Pediatrics | Corewell Health, Helen DeVos Children's Hospital | Grand Rapids, Michigan, USA | ECHO Cohort Study Site Co-Investigator | UG3/UH3OD023348 (Mike O'Shea), UH3OD023347 (Barry Lester) |
| Elizabeth Jensen PhD | Epidemiology and Prevention | Wake Forest University School of Medicine | Winston-Salem, North Carolina, USA | ECHO Cohort Study Site Co-Investigator | UG3/UH3OD023348 (Mike O'Shea) |
| Christina Sakai MD | Pediatrics | Mass General Hospital for Children | Boston, Massachusetts, USA | ECHO Cohort Study Site Co-Investigator | UG3/UH3OD023348 (Mike O'Shea) |
| Hudson Santos RN, PhD | Dean's Office Graduate School, School of Nursing and Health Studies | University of Miami | Coral Gables, Florida, USA | ECHO Cohort Study Site Principal Investigator | UG3/UH3OD023348 (Mike O'Shea), UG3OD035542 (Hudson Santos) |
| Jean M. Kerver PhD, MSc, RD | Departments of Epidemiology & Biostatistics, and Pediatrics & Human Development | Michigan State University, College of Human Medicine | East Lansing, Michigan, USA | ECHO Cohort Study Site Principal Investigator | UG3/UH3OD023285 (Jean Kerver) |
| Nigel Paneth MD, MPH | Departments of Epidemiology & Biostatistics, and Pediatrics & Human Development | Michigan State University, College of Human Medicine | East Lansing, Michigan, USA | ECHO Cohort Study Site Principal Investigator | UG3/UH3OD023285 (Jean Kerver) |
| Charles J. Barone, II MD, FAAP | Department of Pediatrics | Henry Ford Health | Detroit, Michigan, USA | ECHO Cohort Study Site Principal Investigator | UG3/UH3OD023285 (Jean Kerver), UG3/UH3OD023282 (James Gern) |
| Michael R. Elliott PhD | Department of Biostatistics | University of Michigan | Ann Arbor, Michigan, USA | ECHO Cohort Study Site Principal Investigator | UG3/UH3OD023285 (Jean Kerver) |
| Douglas M. Ruden PhD | Department of Obstetrics and Gynecology, Institute of Environmental Health Sciences (IEHS), C.S. Mott Center for Human Health and Development | Wayne State University | Detroit, Michigan, USA | ECHO Cohort Study Site Principal Investigator | UG3/UH3OD023285 (Jean Kerver) |
| Chris Fussman MS | Lifecourse Epidemiology and Genomics Division | Michigan Department of Health and Human Services (MDHHS) | Lansing, Michigan, USA | ECHO Cohort Study Site Principal Investigator | UG3/UH3OD023285 (Jean Kerver) |

| | | | | | |
|---|---|---|---|---|---|
| Julie B. Herbstman PhD | Department of Environmental Health Sciences | Columbia University Mailman School of Public Health | New York, New York, USA | ECHO Cohort Study Site Principal Investigator | UG3/UH3OD023290 (Julie Herbstman) |
| Amy Margolis PhD | Department of Psychiatry | Columbia University Irving Medical Center | New York, New York, USA | ECHO Cohort Study Site Principal Investigator | UG3/UH3OD023290 (Julie Herbstman) |
| Susan L. Schantz PhD | Beckman Institute for Advanced Science and Technology; Department of Comparative Biosciences | University of Illinois Urbana-Champaign | Urbana, Illinois, USA | ECHO Cohort Study Site Principal Investigator | UG3/UH3OD023272 (Susan Schantz) |
| Sarah Dee Geiger PhD | Beckman Institute for Advanced Science and Technology; Department of Kinesiology and Community Health | University of Illinois Urbana-Champaign | Urbana, Illinois, USA | ECHO Cohort Study Site Co-Investigator | UG3/UH3OD023272 (Susan Schantz) |
| Andrea Aguiar PhD | Beckman Institute for Advanced Science and Technology; Department of Comparative Biosciences | University of Illinois Urbana-Champaign | Urbana, Illinois, USA | ECHO Cohort Study Site Co-Investigator | UG3/UH3OD023272 (Susan Schantz) |
| Karen Tabb PhD, MSW | Beckman Institute for Advanced Science and Technology; Department of Social Work | University of Illinois Urbana-Champaign | Urbana, Illinois, USA | ECHO Cohort Study Site Co-Investigator | UG3/UH3OD023272 (Susan Schantz) |
| Rita Strakovsky PhD | Department of Food Science and Human Nutrition | Michigan State University | East Lansing, Michigan, USA | ECHO Cohort Study Site Co-Investigator | UG3/UH3OD023272 (Susan Schantz) |
| Tracey Woodruff PhD, MPH | Program on Reproductive Health and the Environment | University of California, San Francisco | San Francisco, California, USA | ECHO Cohort Study Site Principal Investigator | UG3/UH3OD023272 (Susan Schantz) |
| Rachel Morello-Frosch PhD, MPH | Department of Environmental Science, Policy and Management and School of Public Health | University of California, Berkeley | Berkeley, California, USA | ECHO Cohort Study Site Principal Investigator | UG3/UH3OD023272 (Susan Schantz) |
| Amy Padula PhD | Program on Reproductive Health and the Environment | University of California, San Francisco | San Francisco, California, USA | ECHO Cohort Study Site Co-Investigator | UG3/UH3OD023272 (Susan Schantz) |
| Joseph B. Stanford MD, MSPH | Department of Family and Preventive Medicine | Spencer Fox Eccles School of Medicine, University of Utah | Salt Lake City, Utah, USA | ECHO Cohort Study Site Principal Investigator | UG3/UH3OD023249 (Joseph Stanford) |
| Christina A. Porucznik PhD, MSPH | Department of Family and Preventive Medicine | Spencer Fox Eccles School of Medicine, University of Utah | Salt Lake City, Utah, USA | ECHO Cohort Study Site Principal Investigator | UG3/UH3OD023249 (Joseph Stanford) |
| Angelo P. Giardino MD, PhD | Department of Pediatrics | Spencer Fox Eccles School of Medicine, University of Utah | Salt Lake City, Utah, USA | ECHO Cohort Study Site Principal Investigator | UG3/UH3OD023249 (Joseph Stanford) |
| Rosalind J. Wright MD, MPH | Department of Environmental Medicine & Public Health | Icahn School of Medicine at Mount Sinai | New York, New York, USA | ECHO Cohort Study Site Principal Investigator | UG3/UH3OD023337 (Rosalind Wright) |
| Robert O. Wright MD, MPH | Department of Environmental Medicine & Public Health | Icahn School of Medicine at Mount Sinai | New York, New York, USA | ECHO Cohort Study Site Principal Investigator | UG3/UH3OD023337 (Rosalind Wright) |
| Brent Collett PhD | Department of Psychiatry and Behavioral Medicine | University of Washington, Seattle Children's Research Institute | Seattle, Washington, USA | ECHO Cohort Study Site Principal Investigator | UG3OD035508 (Sheela Sathyanarayana) |
| Nicole Baumann-Blackmore MD | Department of Pediatrics | University of Wisconsin School of Medicine and Public Health | Madison, Wisconsin, USA | ECHO Cohort Study Site Co-Investigator | UG3OD035509 (Anne Marie Singh) |
| Ronald Gangnon PhD | Department of Population Health Sciences | University of Wisconsin | Madison, Wisconsin, USA | ECHO Cohort Study Site Co-Investigator | UG3OD035509 (Anne Marie Singh) |

| | | | | | |
|---|---|---|---|---|---|
| Daniel J. Jackson MD | Department of Pediatrics | University of Wisconsin School of Medicine and Public Health | Madison, Wisconsin, USA | ECHO Cohort Study Site Co-Investigator | UG3OD035509 (Anne Marie Singh) |
| Chris G. McKennan PhD | Department of Statistics | University of Pittsburgh | Pittsburgh, Pennsylvania, USA | ECHO Cohort Study Site Co-Investigator | UG3OD035509 (Anne Marie Singh) |
| Jo Wilson MD | Department of Pediatrics | University of Wisconsin School of Medicine and Public Health | Madison, Wisconsin, USA | ECHO Cohort Study Site Co-Investigator | UG3OD035509 (Anne Marie Singh) |
| Matt Altman MD | Department of Medicine | University of Washington | Seattle, Washington, USA | ECHO Cohort Study Site Co-Investigator | UG3OD035509 (Anne Marie Singh) |
| Judy L. Aschner MD | Department of Pediatrics | Albert Einstein College of Medicine; Hackensack Meridian School of Medicine; Center for Discovery and Innovation | Bronx, New York, USA; Nutley, New Jersey, USA | ECHO Cohort Study Site Principal Investigator | UH3OD023320 and UG3OD035546 (Judy Aschner), UG3OD035513 (Annemarie Stroustrup) |
| Annemarie Stroustrup MD, MPH | Department of Pediatrics | Northwell Health, Cohen Children's Medical Center, and the Zucker School of Medicine at Hofstra / Northwell | New Hyde Park, New York, USA | ECHO Cohort Study Site Principal Investigator | UH3OD023320 (Judy Aschner), UG3OD035513 (Annemarie Stroustrup) |
| Stephanie L. Merhar MD, MS | Department of Pediatrics | Cincinnati Children's | Cincinnati, Ohio, USA | ECHO Cohort Study Site Co-Investigator | UH3OD023320 (Judy Aschner), UG3OD035513 (Annemarie Stroustrup) |
| Paul E. Moore MD | Department of Pediatrics | Vanderbilt University Medical Center | Nashville, Tennessee, USA | ECHO Cohort Study Site Co-Investigator | UH3OD023320 (Judy Aschner), UG3OD035513 (Annemarie Stroustrup) |
| Gloria S. Pryhuber MD | Department of Pediatrics | University of Rochester Medical Center | Rochester, New York, USA | ECHO Cohort Study Site Co-Investigator | UH3OD023320 (Judy Aschner) |
| Mark Hudak MD | Department of Pediatrics | University of Florida College of Medicine | Jacksonville, Florida, USA | ECHO Cohort Study Site Co-Investigator | UH3OD023320 (Judy Aschner) |
| Ann Marie Reynolds Lyndaker MD, MPH | Department of Pediatrics | University of Buffalo Jacobs School of Medicine and Biomedical Sciences | Buffalo, New York, USA | ECHO Cohort Study Site Co-Investigator | UH3OD023320 (Judy Aschner) |
| Andrea L. Lampland MD | Department of Pediatrics | Children's Minnesota | Minneapolis, Minnesota, USA | ECHO Cohort Study Site Co-Investigator | UH3OD023320 (Judy Aschner) |
| Burton Rochelson MD | Department of Obstetrics and Gynecology | Northwell Health and the Zucker School of Medicine at Hofstra / Northwell | New Hyde Park, New York, USA | ECHO Cohort Study Site Principal Investigator | UG3OD035532 (Annemarie Stroustrup) |
| Sophia Jan MD, MSHP | Department of Pediatrics | Northwell Health, Cohen Children's Medical Center, and the Zucker School of Medicine at Hofstra / Northwell | New Hyde Park, New York, USA | ECHO Cohort Study Site Co-Investigator | UG3OD035532 (Annemarie Stroustrup) |
| Matthew J. Blitz MD, MBA | Department of Obstetrics and Gynecology | Northwell Health and the Zucker School of Medicine at Hofstra / Northwell | New Hyde Park, New York, USA | ECHO Cohort Study Site Co-Investigator | UG3OD035532 (Annemarie Stroustrup) |
| Michelle W. Katzow MD, MS | Department of Pediatrics | Northwell Health, Cohen Children's Medical Center, and the Zucker School of Medicine at Hofstra / Northwell | New Hyde Park, New York, USA | ECHO Cohort Study Site Co-Investigator | UG3OD035532 (Annemarie Stroustrup) |

| | | | | | |
|---|---|---|---|---|---|
| Zenobia Brown MD, MPH | Department of Science Education | Northwell Health and the Zucker School of Medicine at Hofstra / Northwell | New Hyde Park, New York, USA | ECHO Cohort Study Site Co-Investigator | UG3OD035532 (Annemarie Stroustrup) |
| Codruta Chiuzan PhD | Institute of Health System Science | Northwell Health, Feinstein Institutes for Medical Research | Manhasset, New York, USA | ECHO Cohort Study Site Co-Investigator | UG3OD035532 (Annemarie Stroustrup) |
| Timothy Rafael MD | Department of Obstetrics and Gynecology | Northwell Health and the Zucker School of Medicine at Hofstra / Northwell | New Hyde Park, New York, USA | ECHO Cohort Study Site Co-Investigator | UG3OD035532 (Annemarie Stroustrup) |
| Dawnette Lewis MD, MPH | Department of Obstetrics and Gynecology | Northwell Health and the Zucker School of Medicine at Hofstra / Northwell | New Hyde Park, New York, USA | ECHO Cohort Study Site Co-Investigator | UG3OD035532 (Annemarie Stroustrup) |
| Natalie Meirowitz MD | Department of Obstetrics and Gynecology | Northwell Health and the Zucker School of Medicine at Hofstra / Northwell | New Hyde Park, New York, USA | ECHO Cohort Study Site Co-Investigator | UG3OD035532 (Annemarie Stroustrup) |
| Brenda Poindexter MD | Department of Pediatrics | Children's Healthcare of Atlanta Emory University | Atlanta, Georgia, USA | ECHO Cohort Study Site Co-Investigator | UH3OD023320 (Judy Aschner) |
| Tebeb Gebretsadik MPH | Department of Biostatistics | Vanderbilt University Medical Center | Nashville, Tennessee, USA | ECHO Cohort Study Site Principal Investigator | UG3OD035516 and UG3OD035517 (Tina Hartert) |
| Sarah Osmundson MD, MSC | Department of Obstetrics and Gynecology | Vanderbilt University Medical Center | Nashville, Tennessee, USA | ECHO Cohort Study Site Principal Investigator | UG3OD035517 (Tina Hartert) |
| Jennifer K. Straughen PhD | Department of Public Health Sciences | Henry Ford Health | Detroit, Michigan, USA | ECHO Cohort Study Site Principal Investigator | UG3OD035518 (Jennifer Straughen) |
| Amy Eapen MD | Division of Allergy and Clinical Immunology | Henry Ford Health | Detroit, Michigan, USA | ECHO Cohort Study Site Principal Investigator | UG3OD035518 (Jennifer Straughen) |
| Andrea Cassidy-Bushrow PhD | Department of Public Health Sciences | Henry Ford Health | Detroit, Michigan, USA | ECHO Cohort Study Site Co-Investigator | UG3/UH3OD023282 (James Gern) |
| Ganesa Wegienka PhD | Department of Public Health Sciences | Henry Ford Health | Detroit, Michigan, USA | ECHO Cohort Study Site Co-Investigator | UG3/UH3OD023282 (James Gern) |
| Alex Sitarik MPH | Department of Public Health Sciences | Henry Ford Health | Detroit, Michigan, USA | ECHO Cohort Study Site Biostatistician | UG3/UH3OD023282 (James Gern) |
| Kim Woodcroft PhD | Department of Public Health Sciences | Henry Ford Health | Detroit, Michigan, USA | ECHO Cohort Study Site Co-Investigator | UG3OD035518 (Jennifer Straughen), UG3/UH3OD023282 (James Gern) |
| Audrey Urquhart MPH | Department of Public Health Sciences | Henry Ford Health | Detroit, Michigan, USA | ECHO Cohort Study Site Epidemiologist | UG3OD035518 (Jennifer Straughen), UG3/UH3OD023282 (James Gern) |
| Albert Levin PhD | Department of Public Health Sciences | Henry Ford Health | Detroit, Michigan, USA | ECHO Cohort Study Site Co-Investigator | UG3OD035518 (Jennifer Straughen) |
| Tisa Johnson-Hooper MD | Department of Pediatrics | Henry Ford Health | Detroit, Michigan, USA | ECHO Cohort Study Site Co-Investigator | UG3OD035518 (Jennifer Straughen) |
| Brent Davidson MD | Department of Women's Health | Henry Ford Health | Detroit, Michigan, USA | ECHO Cohort Study Site Co-Investigator | UG3/UH3OD023282 (James Gern) |
| Tengfei Ma PhD | Department of Public Health Sciences | Henry Ford Health | Detroit, Michigan, USA | ECHO Cohort Study Site Co-Investigator | UG3OD035518 (Jennifer Straughen) |

| | | | | | |
|---|---|---|---|---|---|
| Emily S. Barrett PhD | Department of Biostatistics and Epidemiology | Environmental and Occupational Health Sciences Institute, Rutgers University | Piscataway, New Jersey, USA | ECHO Cohort Study Site Principal Investigator | UG3OD035527 (Emily S Barrett) |
| Martin J. Blaser MD | Center for Advanced Biotechnology & Medicine | Rutgers University | Piscataway, New Jersey, USA | ECHO Cohort Study Site Principal Investigator | UG3OD035527 (Emily S Barrett) |
| Maria Gloria Dominguez-Bello PhD | Departments of Biochemistry and Microbiology & Anthropology | Rutgers University | New Brunswick, New Jersey, USA | ECHO Cohort Study Site Principal Investigator | UG3OD035527 (Emily S Barrett) |
| Daniel B. Horton MD | Department of Pediatrics | Robert Wood Johnson Medical School, Rutgers University | New Brunswick, New Jersey, USA | ECHO Cohort Study Site Principal Investigator | UG3OD035527 (Emily S Barrett) |
| Manuel Jimenez MD | Departments of Pediatrics, Family Medicine, and Community Health | Robert Wood Johnson Medical School, Rutgers University | New Brunswick, New Jersey, USA | ECHO Cohort Study Site Principal Investigator | UG3OD035527 (Emily S Barrett) |
| Todd Rosen MD | Department of Obstetrics, Gynecology, and Reproductive Sciences | Robert Wood Johnson Medical School, Rutgers University | New Brunswick, New Jersey, USA | ECHO Cohort Study Site Co-Investigator | UG3OD035527 (Emily S Barrett) |
| Kristy Palomares MD, PhD | Department of Obstetrics and Gynecology | Saint Peter's University Hospital | New Brunswick, New Jersey, USA | ECHO Cohort Study Site Co-Investigator | UG3OD035527 (Emily S Barrett) |
| Lyndsay A. Avalos PhD, MPH | Division of Research | Kaiser Permanente Northern California | Oakland, California, USA | ECHO Cohort Study Site Principal Investigator | UG3OD035540 (Monique Marie Hedderson) |
| Yeyi Zhu PhD, MS | Division of Research | Kaiser Permanente Northern California | Oakland, California, USA | ECHO Cohort Study Site Principal Investigator | UG3OD035540 (Monique Marie Hedderson) |
| Kelly J . Hunt PhD | Department of Public Health Sciences | Medical University of South Carolina | Charleston, South Carolina, USA | ECHO Cohort Study Site Principal Investigator | UG3OD035543 (Kelly J Hunt) |
| Roger B. Newman MD | Department of Obstetrics and Gynecology | Medical University of South Carolina | Charleston, South Carolina, USA | ECHO Cohort Study Site Principal Investigator | UG3OD035543 (Kelly J Hunt) |
| Michael S. Bloom PhD | Department of Global and Community Health | George Mason University | Fairfax, Virginia, USA | ECHO Cohort Study Site Principal Investigator | UG3OD035543 (Kelly J Hunt) |
| Mallory H. Alkis MD | Department of Obstetrics and Gynecology | Medical University of South Carolina | Charleston, South Carolina, USA | ECHO Cohort Study Site Co-Investigator | UG3OD035543 (Kelly J Hunt) |
| James R. Roberts MD, MPH | Department of Pediatrics | Medical University of South Carolina | Charleston, South Carolina, USA | ECHO Cohort Study Site Co-Investigator | UG3OD035543 (Kelly J Hunt) |
| Sunni L. Mumford PhD | Department of Biostatistics, Epidemiology and Informatics; Department of Obstetrics and Gynecology | University of Pennsylvania Perelman School of Medicine | Philadelphia, Pennsylvania, USA | ECHO Cohort Study Site Principal Investigator | UG3OD035537 (Sunni L Mumford) |
| Heather H. Burris MD, MPH | Division of Neonatology, Department of Pediatrics | Children's Hospital of Philadelphia; University of Pennsylvania Perelman School of Medicine | Philadelphia, Pennsylvania, USA | ECHO Cohort Study Site Principal Investigator | UG3OD035537 (Sunni L Mumford) |
| Sara B. DeMauro MD, MSCE | Division of Neonatology, Department of Pediatrics | Children's Hospital of Philadelphia; University of Pennsylvania Perelman School of Medicine | Philadelphia, Pennsylvania, USA | ECHO Cohort Study Site Principal Investigator | UG3OD035537 (Sunni L Mumford) |
| Lynn M. Yee MD, MPH | Division of Maternal-Fetal Medicine, Department of Obstetrics & Gynecology | Feinberg School of Medicine, Northwestern University | Chicago, Illinois, USA | ECHO Cohort Study Site Principal Investigator | UG3OD035546 (Judy Aschner) |

| | | | | | |
|---|---|---|---|---|---|
| Aaron Hamvas MD | Division of Neonatology, Department of Pediatrics | Ann & Robert H. Lurie Children's Hospital, Feinberg School of Medicine, Northwestern University | Chicago, Illinois, USA | ECHO Cohort Study Site Principal Investigator | UG3OD035546 (Judy Aschner) |
| Antonia F. Olidipo MD, MSCI | Division of Maternal-Fetal Medicine, Department of Obstetrics & Gynecology | Hackensack University Medical Center, Hackensack Meridian School of Medicine | Nutley, New Jersey, USA | ECHO Cohort Study Site Co-Investigator | UG3OD035546 (Judy Aschner) |
| Andrew S. Haddad MD | Division of Maternal-Fetal Medicine, Department of Obstetrics & Gynecology | Hackensack University Medical Center, Hackensack Meridian School of Medicine | Nutley, New Jersey, USA | ECHO Cohort Study Site Co-Investigator | UG3OD035546 (Judy Aschner) |
| Lisa R. Eiland MD | Division of Neonatology, Department of Pediatrics | Hackensack University Medical Center, Hackensack Meridian School of Medicine | Nutley, New Jersey, USA | ECHO Cohort Study Site Co-Investigator | UG3OD035546 (Judy Aschner) |
| Nicole T. Spillane MD | Division of Neonatology, Department of Pediatrics | Hackensack University Medical Center, Hackensack Meridian School of Medicine | Nutley, New Jersey, USA | ECHO Cohort Study Site Co-Investigator | UG3OD035546 (Judy Aschner) |
| Kirin N. Suri MD | Division of Developmental and Behavioral Pediatrics, Department of Pediatrics | Hackensack University Medical Center, Hackensack Meridian School of Medicine | Nutley, New Jersey, USA | ECHO Cohort Study Site Co-Investigator | UG3OD035546 (Judy Aschner) |
| Stephanie A. Fisher MD, MPH | Division of Maternal-Fetal Medicine, Department of Obstetrics & Gynecology | Feinberg School of Medicine, Northwestern University | Chicago, Illinois, USA | ECHO Cohort Study Site Co-Investigator | UG3OD035546 (Judy Aschner) |
| Jeffrey A. Goldstein MD, PhD | Department of Pathology | Feinberg School of Medicine, Northwestern University | Chicago, Illinois, USA | ECHO Cohort Study Site Co-Investigator | UG3OD035546 (Judy Aschner) |
| Leena B. Mithal MD | Division of Infectious Diseases, Department of Pediatrics | Ann & Robert H. Lurie Children's Hospital, Feinberg School of Medicine, Northwestern University | Chicago, Illinois, USA | ECHO Cohort Study Site Co-Investigator | UG3OD035546 (Judy Aschner) |
| Raye-Ann O. DeRegnier MD | Division of Neonatology, Department of Pediatrics | Ann & Robert H. Lurie Children's Hospital, Feinberg School of Medicine, Northwestern University | Chicago, Illinois, USA | ECHO Cohort Study Site Co-Investigator | UG3OD035546 (Judy Aschner) |
| Nathalie L. Maitre MD, PhD | Division of Neonatology, Department of Pediatrics | Emory University School of Medicine and Cerebral Palsy Foundation | Atlanta, Georgia, USA and New York, New York, USA | ECHO Cohort Study Site Co-Investigator | UG3OD035546 (Judy Aschner) |
| Ruby H.N. Nguyen PhD, MHS | Division of Epidemiology & Community Health | School of Public Health, University of Minnesota | Minneapolis, Minnesota, USA | ECHO award Principal Investigator | UG3OD035529 (Hong-Ngoc Nguyen) |
| Meghan M. JaKa PhD, MS | Division of Research & Evaluation | HealthPartners Institute | Minneapolis, Minnesota, USA | ECHO site Principal Investigator | UG3OD035529 (Hong-Ngoc Nguyen) |
| Abbey C. Sidebottom PhD, MPH | Care Delivery Research | Allina Health | Minneapolis, Minnesota, USA | ECHO site Principal Investigator | UG3OD035529 (Hong-Ngoc Nguyen) |

| | | | | | |
|---|---|---|---|---|---|
| Michael J. Paidas MD | Department of Obstetrics and Gynecology | University of Miami Miller School of Medicine | Miami, Florida, USA | ECHO site Principal Investigator | UG3OD035542 (Hudson Santos) |
| JoNell E. Potter APRN, PhD | Department of Obstetrics, Gynecology and Reproductive Sciences | University of Miami Miller School of Medicine | Miami, Florida, USA | ECHO Cohort Study Site Co-Investigator | UG3OD035542 (Hudson Santos) |
| Natale Ruby PhD, PsyD | Mailman Center for Child Development | University of Miami Miller School of Medicine | Miami, Florida, USA | ECHO Cohort Study Site Co-Investigator | UG3OD035542 (Hudson Santos) |
| Lunthita Duthely EdD | Department of Obstetrics, Gynecology and Reproductive Sciences and Department of Public Health Sciences | University of Miami School of Medicine | Miami, Florida, USA | ECHO Cohort Study Site Co-Investigator | UG3OD035542 (Hudson Santos) |
| Arumugam Jayakumar PhD | Department of Obstetrics, Gynecology and Reproductive Sciences | University of Miami Miller School of Medicine | Miami, Florida, USA | ECHO Cohort Study Site Co-Investigator | UG3OD035542 (Hudson Santos) |
| Karen Young MD | Department of Pediatrics | University of Miami Miller School of Medicine | Miami, Florida, USA | ECHO Cohort Study Site Co-Investigator | UG3OD035542 (Hudson Santos) |
| Isabel Maldonado MPH, BS | School of Nursing and Health Studies | University of Miami | Miami, Florida, USA | ECHO Cohort Study Site Program Director | UG3OD035542 (Hudson Santos) |
| Meghan Miller PhD | Psychiatry and Behavioral Sciences; MIND Institute | University of California Davis | Sacramento, California, USA | ECHO Cohort Study Site Co-Investigator | UG3OD035550 (Rebecca Schmidt) |
| Jonathan L. Slaughter MD, MPH | Center for Perinatal Research, Abigail Wexner Research Institute and Division of Neonatology, Nationwide Children's Hospital and Department of Pediatrics, College of Medicine and Division of Epidemiology, College of Public Health, The Ohio State University | Nationwide Children's Hospital and The Ohio State University | Columbus, Ohio, USA | ECHO Cohort Study Site Principal Investigator | UG3OD035536 (Jonathan Slaughter) |
| Sarah A. Keim PhD, MS, MA | Center for Biobehavioral Health, Abigail Wexner Research Institute, Nationwide Children's Hospital and Department of Pediatrics, College of Medicine and Division of Epidemiology, College of Public Health, The Ohio State University | Nationwide Children's Hospital and The Ohio State University | Columbus, Ohio, USA | ECHO Cohort Study Site Principal Investigator | UG3OD035536 (Jonathan Slaughter) |
| Courtney D. Lynch PhD, MPH | Division of Maternal-Fetal Medicine, Department of Obstetrics and Gynecology, College of Medicine and Division of Epidemiology, College of Public Health, The Ohio State University | The Ohio State University | Columbus, Ohio, USA | ECHO Cohort Study Site Principal Investigator | UG3OD035536 (Jonathan Slaughter) |
| Kartik K. Venkatesh MD, PhD | Division of Maternal-Fetal Medicine, Department of Obstetrics and Gynecology, College of Medicine and Division of Epidemiology, College of Public Health, The Ohio State University | The Ohio State University | Columbus, Ohio, USA | ECHO Cohort Study Site Principal Investigator | UG3OD035536 (Jonathan Slaughter) |
| Kristina W. Whitworth PhD | Center for Precision Environmental Health and Department of Medicine | Baylor College of Medicine | Houston, Texas, USA | ECHO Cohort Study Site Principal Investigator | UG3OD035544 (Kristina Whitworth) |

| | | | | | |
|---|---|---|---|---|---|
| Elaine Symanski PhD | Center for Precision Environmental Health and Department of Medicine | Baylor College of Medicine | Houston, Texas, USA | ECHO Cohort Study Site Principal Investigator | UG3OD035544 (Kristina Whitworth) |
| Thomas F. Northrup PhD | Department of Family and Community Medicine | University of Texas Health Science Center at Houston (UTHealth Houston) McGovern Medical School | Houston, Texas, USA | ECHO Cohort Study Site Principal Investigator | UG3OD035544 (Kristina Whitworth) |
| Hector Mendez-Figueroa MD | Department of Obstetrics, Gynecology and Reproductive Sciences | University of Texas Health Science Center at Houston (UTHealth Houston) McGovern Medical School | Houston, Texas, USA | ECHO Cohort Study Site Co-Investigator | UG3OD035544 (Kristina Whitworth) |
| Ricardo A. Mosquera MD | Department of Pediatrics | University of Texas Health Science Center at Houston (UTHealth Houston) McGovern Medical School | Houston, Texas, USA | ECHO Cohort Study Site Co-Investigator | UG3OD035544 (Kristina Whitworth) |
| Margaret R. Karagas PhD | Department of Epidemiology | Geisel School of Medicine at Dartmouth | Hanover, New Hampshire, USA | ECHO Cohort Study Site Principal Investigator | UG3/UH3OD023275 (Margaret Karagas) |
| Juliette C. Madan MD, MS | Departments of Psychiatry, Pediatrics & Epidemiology | Geisel School of Medicine at Dartmouth, Dartmouth Hitchcock Medical Center | Hanover, New Hampshire, USA | ECHO Cohort Study Site Principal Investigator | UG3/UH3OD023275 (Margaret Karagas) |
| Debra M. MacKenzie PhD | Community Environmental Health Program, Department of Pharmaceutical Sciences | College of Pharmacy, University of New Mexico Health Sciences Center | Albuquerque, New Mexico, USA | ECHO Cohort Study Site Principal Investigator | UG3/UH3OD023344 (Debra MacKenzie) |
| Johnnye L. Lewis PhD | Community Environmental Health Program, Department of Pharmaceutical Sciences | College of Pharmacy, University of New Mexico Health Sciences Center | Albuquerque, New Mexico, USA | ECHO Cohort Study Site Principal Investigator | UG3/UH3OD023344 (Debra MacKenzie) |
| Brandon J. Rennie PhD | Center for Development and Disability | University of New Mexico | Albuquerque, New Mexico, USA | ECHO Cohort Study Site Co-Investigator | UG3/UH3OD023344 (Debra MacKenzie) |
| Bennett L. Leventhal MD | Community Environmental Health Program, Department of Pharmaceutical Sciences UNM | College of Pharmacy, University of New Mexico Health Sciences Center; University of Chicago | Albuquerque, New Mexico, USA; Chicago, Illinois, USA | ECHO Cohort Study Site Co-Investigator | UG3/UH3OD023344 (Debra MacKenzie) |
| Young Shin Kim MD, MS, MPH, PhD | Department of Psychiatry and Behavioral Sciences | University of California, San Francisco | San Francisco, California, USA | ECHO Cohort Study Site Co-Investigator | UG3/UH3OD023344 (Debra MacKenzie) |
| Somer Bishop PhD | Department of Psychiatry and Behavioral Sciences | University of California, San Francisco | San Francisco, California, USA | ECHO Cohort Study Site Co-Investigator | UG3/UH3OD023344 (Debra MacKenzie) |
| Sara S. Nozadi PhD | Community Environmental Health Program, Department of Pharmaceutical Sciences | College of Pharmacy, University of New Mexico Health Sciences Center | Albuquerque, New Mexico, USA | ECHO Cohort Study Site Co-Investigator | UG3/UH3OD023344 (Debra MacKenzie) |
| Li Luo PhD | Department of Internal Medicine | Comprehensive Cancer Center, University of New Mexico Health Sciences Center | Albuquerque, New Mexico, USA | ECHO Cohort Study Site Co-Investigator | UG3/UH3OD023344 (Debra MacKenzie) |
| Barry M. Lester PhD | Department of Pediatrics, Department of Psychiatry and Human Behavior | Warren Alpert Medical School of Brown University | Providence, Rhode Island, USA | ECHO Cohort Study Site Principal Investigator | UH3OD023347 (Barry Lester) |
| Carmen J. Marsit PhD | Department of Environmental Health | Rollins School of Public Health, Emory University | Atlanta, Georgia, USA | ECHO Cohort Study Site Principal Investigator | UH3OD023347 (Barry Lester) |
| Todd Everson PhD | Department of Environmental Health | Rollins School of Public Health, Emory University | Atlanta, Georgia, USA | ECHO Cohort Study Site Principal Investigator | UH3OD023347 (Barry Lester) |
| Cynthia M. Loncar PhD | Department of Psychiatry and Human Behavior | Warren Alpert Medical School of Brown University | Providence, Rhode Island, USA | ECHO Cohort Study Site Principal Investigator | UH3OD023347 (Barry Lester) |

| | | | | | |
|---|---|---|---|---|---|
| Elisabeth C. McGowan MD | Department of Pediatrics | Warren Alpert Medical School of Brown University | Providence, Rhode Island, USA | ECHO Cohort Study Site Principal Investigator | UH3OD023347 (Barry Lester) |
| Stephen J. Sheinkopf PhD | Department of Pediatrics | Thompson Center for Autism & Neurodevelopment, University of Missouri | Columbia, Missouri, USA | ECHO Cohort Study Site Principal Investigator | UH3OD023347 (Barry Lester) |
| Brian S. Carter MD | Department of Pediatrics | Children's Mercy-Kansas City | Kansas City, Missouri, USA | ECHO Cohort Study Site Principal Investigator | UH3OD023347 (Barry Lester) |
| Jennifer Check MD | Department of Pediatrics | Wake Forest School of Medicine | Winston, Salem North Carolina, USA | ECHO Cohort Study Site Principal Investigator | UH3OD023347 (Barry Lester) |
| Jennifer B. Helderman MD | Department of Pediatrics | Wake Forest School of Medicine | Winston, Salem North Carolina, USA | ECHO Cohort Study Site Principal Investigator | UH3OD023347 (Barry Lester) |
| Charles R. Neal MD | Department of Pediatrics | University of Hawaii John A Burns School of Medicine | Honolulu, Hawaii, USA | ECHO Cohort Study Site Principal Investigator | UH3OD023347 (Barry Lester) |
| Lynne M. Smith MD | Department of Pediatrics | UCLA Clinical and Translational Science Institute at The Lundquist Institute, Harbor-UCLA Medical Center | Los Angeles, California, USA | ECHO Cohort Study Site Principal Investigator | UH3OD023347 (Barry Lester) |
